# RcsB regulation of the YfdX-mediated acid stress response in *Klebsiella pneumoniae* CG43S3

**DOI:** 10.1371/journal.pone.0212909

**Published:** 2019-02-28

**Authors:** Chia-Jui Liu, Ching-Ting Lin, Jo-Di Chiang, Chen-Yi Lin, Yen-Xi Tay, Li-Cheng Fan, Kuan-Nan Peng, Chih-Huan Lin, Hwei-Ling Peng

**Affiliations:** 1 Department of Biological Science and Technology, School of Biological Science and Technology, National Chiao Tung University, Hsinchu, Taiwan, Republic of China; 2 School of Chinese Medicine, China Medical University, Taichung, Taiwan, Republic of China; 3 Institute of Molecular Medicine and Biological Technology, School of Biological Science and Technology, National Chiao Tung University, Hsinchu, Taiwan, Republic of China; University of British Columbia, CANADA

## Abstract

In *Klebsiella pneumoniae* CG43S3, deletion of the response regulator gene *rcsB* reduced the capsular polysaccharide amount and survival on exposure to acid stress. A comparison of the pH 4.4-induced proteomes between CG43S3 and CG43S3Δ*rcsB* revealed numerous differentially expressed proteins and one of them, YfdX, which has recently been reported as a periplasmic protein, was absent in CG43S3Δ*rcsB*. Acid survival analysis was then conducted to determine its role in the acid stress response. Deletion of *yfdX* increased the sensitivity of *K*. *pneumoniae* CG43S3 to a pH of 2.5, and transforming the mutant with a plasmid carrying *yfdX* restored the acid resistance (AR) levels. In addition, the effect of *yfdX* deletion was cross-complemented by the expression of the periplasmic chaperone HdeA. Furthermore, the purified recombinant protein YfdX reduced the acid-induced protein aggregation, suggesting that YfdX as well as HdeA functions as a chaperone. The following promoter activity measurement revealed that *rcsB* deletion reduced the expression of *yfdX* after the bacteria were subjected to pH 4.4 adaptation. Western blot analysis also revealed that YfdX production was inhibited by *rcsB* deletion and only the plasmid expressing RcsB or the nonphosphorylated form of RcsB, RcsB_D56A_, could restore the YfdX production, and the RcsB-mediated complementation was no longer observed when the sensor kinase RcsD gene was deleted. In conclusion, this is the first study demonstrating that YfdX may be involved in the acid stress response as a periplasmic chaperone and that RcsB positively regulates the acid stress response partly through activation of *yfdX* expression. Moreover, the phosphorylation status of RcsB may affect the YfdX expression under acidic conditions.

## Introduction

The nosocomial pathogen *Klebsiella pneumoniae* causes suppurative lesions, septicemia, and infections of the urinary and respiratory tracts in immunocompromised patients [1, 2]. In Taiwan, the incidence of *Klebsiella* liver abscesses (KLAs) in patients with diabetes, malignancies, renal diseases, and pneumonia has steadily increased [3]. Recently, KLAs have also been reported in Western and other Asian countries [4]. Although several virulence traits, including K1 capsular polysaccharides [3], *magA* [5], iron acquisition loci on pLVPK [6], and type 1 and type 3 fimbriae [7, 8], have been implicated in the pathogenesis of KLAs, the pathogenic mechanism underlying KLAs remains unknown. The endogenous *K*. *pneumoniae* residing in a patient’s gastrointestinal (GI) tract has been reported to be the predisposing factor for KLA and several gastrointestinal diseases [9–11]. In addition, a recent report indicated that hospital-acquired *K*. *pneumoniae* infections are largely associated with the patients’ own GI microbiota [12]. Conceivably, determining the mechanism by which *K*. *pneumoniae* is retained in the GI tract is essential to elucidate the pathogenic mechanism. During GI colonization, exposure to acid pH in the stomach is a challenge that the bacteria must overcome. In *K*. *pneumoniae*, the tripartite efflux pump EffABC, lysine decarboxylase operon *cadCBA* and OxyR have been reported to regulate resistance to HCl [13–15].

**Fig 1:**
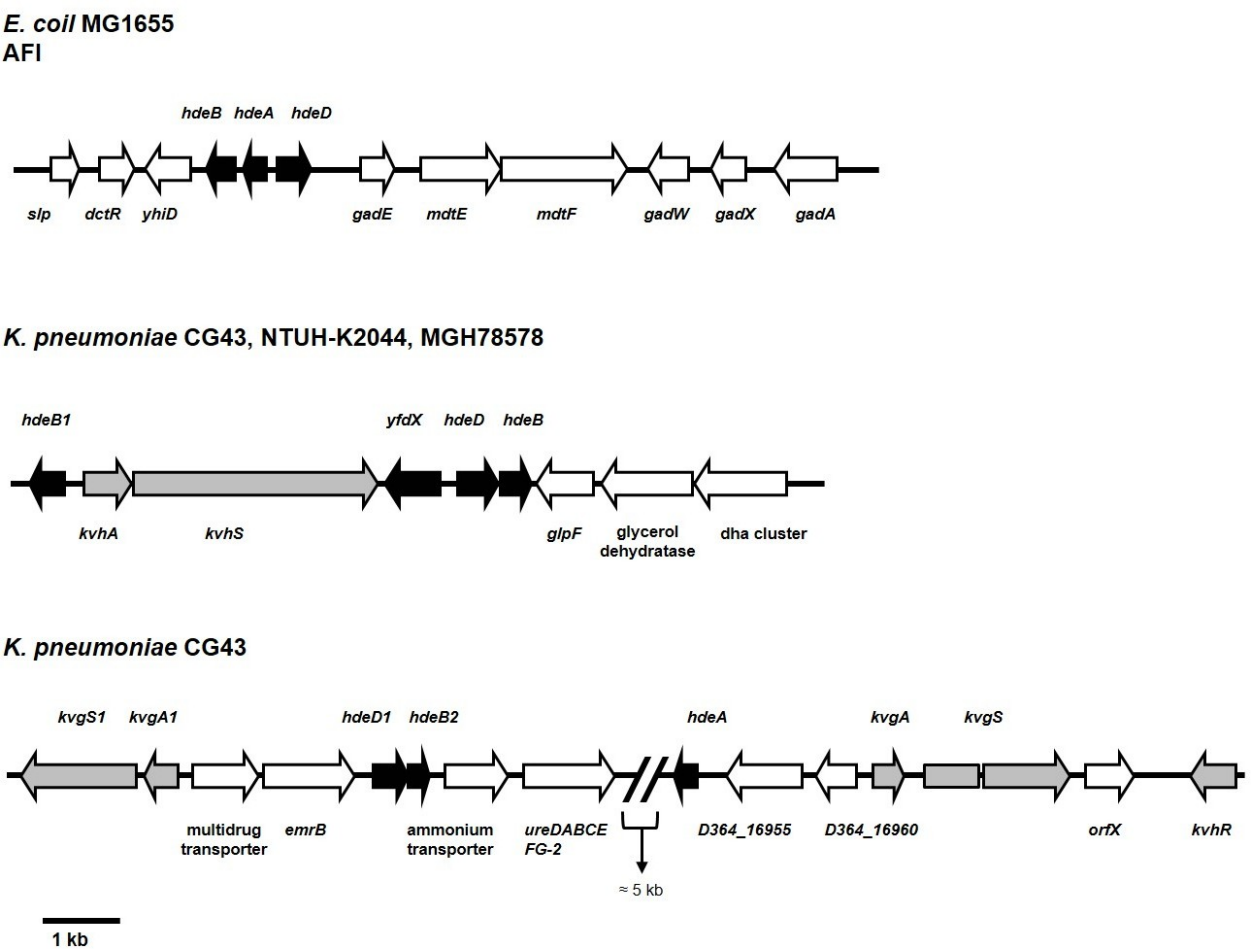
Organization of the AFI gene cluster and *yfdX* in *E*. *coli* MG1655 and *K*. *pneumoniae* CG43, NTUH-K2044, and MGH78578. The genes were annotated according to the National Center for Biotechnology Information (Version 3.20.3) using BLASTX analysis. In *E*. *coli* MG1655, the cluster of genes from *slp* to *gadA* has been designated as an acid fitness island (AFI). The genes *yfdX*, *hdeDB*, and *hdeB1* were found in all three *K*. *pneumoniae* genomes, whereas *hdeA* was identified only in the CG43 genome. The cluster of genes from *kvgS1* to *kvhR* was found in CG43 genome but not in the genome of NTUH-K2044 and MGH78578.

AR is a crucial adaptation in enterobacteria for tolerating stomach acids before intestinal tract colonization. *Escherichia coli* has five AR systems, AR1–AR5, which enable it to survive in acidic environments [16]. In extremely acidic environments (pH 2.5), the glutamate-dependent system AR2 is activated and then the decarboxylation of glutamic acid depletes intracellular protons, thereby increasing the pH of the cytoplasm [16]. In addition to the AR system, the acid fitness island (AFI), which consists of 12 genes including *gadAEWX*, *mdtEF*, *slp*, *dctR*, *yhiD*, and *hdeABD*, plays a role in the acid response [17]. As shown in Fig 1, the *hdeAB* operon and *hdeD* are divergently transcribed, but they all encode a periplasmic chaperone. Studies have shown that HdeA functions under extremely acidic conditions (pH lower than 3), whereas HdeB functions optimally between pH 4 and 5 [18–20]. The *hdeAB* operon has also been identified in *Shigella flexneri* and *Brucella abortus*, but not in *Salmonella typhimurium* and *Vibrio cholera* [21]. Although few studies have focused on HdeD, it has been shown to play a role in high-cell-density AR [17].

Different from *E*. *coli*, a sequence search for the genomes of the *K*. *pneumoniae* clinical isolates MGH78578 [22], NTUH-K2044 [23], and CG43 (NCBI Taxonomy ID: 1244085) revealed no AR2 and AR3 encoding genes. Nevertheless, *hdeB* and *hdeD* were clustered with the two-component system (TCS) coding genes *kvhAS* [24–26] and *hdeB1* (Fig 1). Notably, although not identified in the genome of MGH78578 and NTUH-K2044, *hdeA* was found next to *kvgAS* [24] and a distantly located homolog of *hdeDB* (designated as *hdeD1B2*) in the CG43 genome (Fig 1). *K*. *pneumoniae* CG43 and NTUH-K2044 are heavily encapsulated liver abscess isolates and have recently been classified as hypermucoviscosity strains [23, 27]. Given that HdeA may confer CG43 a higher AR activity than NTUH-K2044, we compared the acid stress survivals between CG43S3 and NTUH-K2044S3. However, S1 Fig revealed that NTUH-K2044S3 exhibited a stronger AR than CG43S3 at either the exponential or stationary phase. The TCS KvgAS and KvhAS, homologs of *E*. *coli* EvgAS, have previously been reported to be involved in the regulation of the virulence, drug resistance, stress response and capsular polysaccharide biosynthesis [24–26]. A possibility of KvhAS regulation on the expression of *yfdX* and *hdeDB* has also been speculated [25].

Bacteria are equipped with a complex regulatory pathway to ensure an appropriate and rapid response to environmental acid stress. In *E*. *coli*, the TCS EvgAS is a major determinant in the regulation of the acid stress response and multidrug resistance [28–30]. The RcsBCD TCS also plays a role in the acid stress response through the regulation of AR2 expression. In the absence of GadE, RcsB represses the expression of *gadABC*, whereas it activates the expression of the Gad operon in the presence of GadE by forming an RcsB–GadE heterodimer [31–34]. In addition, the deletion of *rcsB* in *E*. *coli* O157:H7 impairs the expression of HdeA, which leads to increased acid sensitivity [35, 36].

The Rcs system is composed of three core proteins, innermembrane sensor kinase RcsC, phosphotransferase RcsD, and response regulator RcsB. In addition, the outermembrane lipoprotein RcsF is an auxiliary protein for receiving extracellular stimulations. The signal transduction of Rcs phosphorelay begins with the autophosphorylation of RcsC upon receiving specific stimuli, and then RcsD transfers the phosphoryl group from RcsC to the cytoplasmic regulator RcsB [37]. The phosphoryl group is transferred to the conserved aspartate residue (D56) of RcsB, and the phosphorylation status of RcsB determines its regulatory property [38, 39].

In *K*. *pneumoniae*, apart from playing a role in regulating capsule production [40, 41], RcsB has also been reported to play a role in resistance to polymyxin B [42, 43]. In this study, we investigated the involvement of RcsB in regulating the acid stress response in *K*. *pneumoniae* CG43S3 by analyzing the deletion effect of the *rcs* system on the bacterial resistance to acid stress and used a comparative proteomic approach to identify the downstream genes regulated by RcsB.

## Materials and methods

### Bacterial strains, plasmid, primer and growth conditions

Table 1 lists the bacterial strains and plasmids and Table 2 lists the primers used in this study. Bacteria were grown in Luria-Bertani [44] broth at 37°C. The antibiotics used included ampicillin (100 μg/ml), kanamycin (25 μg/ml), streptomycin (500 μg/ml), and chloramphenicol (35 μg/ml).

**Table 1:** Bacterial strains and plasmids used in this study.

Strain or plasmid	Properties ^a^	Reference or source
***K*. *pneumoniae* Strains**		
NTUH-K2044	K1 serotype, hypermucoviscosity	[23]
CG43S3	CG43 derived strain, *rspL* mutant (Sm^r^)	[40]
CG43S3Δ*rcsB*	CG43S3 with deletion of *rcsB* gene	[40]
CG43S3Δ*rcsC*	CG43S3 with deletion of *rcsC* gene	This study
CG43S3Δ*rcsD*	CG43S3 with deletion of *rcsD* gene	This study
CG43S3Δ*rcsF*	CG43S3 with deletion of *rcsF* gene	This study
CG43S3Δ*yfdX*	CG43S3 with deletion of *yfdX* gene	This study
CG43S3Δ*hdeB*	CG43S3 with deletion of *hdeB* genes	This study
CG43S3Δ*hdeB1*	CG43S3 with deletion of *hdeB1* gene	This study
CG43S3Δ*hdeB2*	CG43S3 with deletion of *hdeB2* gene	This study
CG43S3Δ*hdeA*	CG43S3 with deletion of *hdeA* gene	This study
CG43S3Δ*hdeD*	CG43S3 with deletion of *hdeD* gene	This study
CG43S3Δ*hdeD1*Δ*hdeB2*	CG43S3 with deletion of *hdeD1*, *hdeB2* genes	This study
CG43S3Δ*hns*	CG43S3 with deletion of *hns* gene	This study
CG43S3Δ*lacZ*	CG43S3 with deletion *lacZ* genes	[25]
CG43S3Δ*lacZ*Δ*rcsB*	CG43S3Δ*rcsB* with deletion of *lacZ* gene	This study
***E*. *coli* Strains**		
JM109	Cloning host, *recA1*, *supE44*, *endA1*, *hsdR17*, *gyrA96*(NalR), *relA1*, *thi-1*, Δ(*lac-proAB*) /F´ [*traD36*, *proAB*, *laqI*^q^*Z*ΔM15]	Laboratory stock
S17-1λ*pir*	Bacterial conjugation, Tp^r^ Sm^r^ *recA*, *thi*, *pro*, *hsdR*^-^*M*^+^[PR4-2-Tc::Mu:Km^r^ Tn7](*pir*)	Laboratory stock
NovaBlue (DE3)	Recombinant protein overexpression host	Laboratory stock
**Plasmids**		
pKAS46	suicide vector, Km^r^ Ap^r^	[45]
yT&A	cloning vector, Ap^r^	Yeastern Biotech
pET30a	His-tag fusion protein expression vector, Km^r^	Novagen
pET30a-*rcsB*	*rcsB* coding region cloned into pET30a, Km^r^	This study
pET30a-*yfdX*	*yfdX* coding region cloned into pET30a, Km^r^	This study
pRK415	Broad-host-range IncP plasmid, Tc^r^	[46]
pRK415-*yfdX*	*yfdX* complement plasmid, Tc^r^	This study
pRK415-*hdeA*	*hdeA* complement plasmid, Tc^r^	This study
pRK415-*hdeB*	*hdeB* complement plasmid, Tc^r^	This study
pRK415-*hdeD*	*hdeD* complement plasmid, Tc^r^	This study
pRK415-*hdeDB*	*hdeB* and *hdeD* complement plasmid, Tc^r^	This study
pRK415-*hdeA*-F44A	*hdeA* F44A complement plasmid, Tc^r^	This study
pRK415-*rcsB*	*rcsB* complement plasmid, Tc^r^	This study
pRK415-*rcsB*-D56A	*rcsB* D56A complement plasmid, Tc^r^	This study
pRK415-*rcsB*-D56E	*rcsB* D56E complement plasmid, Tc^r^	This study
pLacZ15	A derivative of pYC016, containing a promoterless *lacZ* from *K*. *pneumoniae* CG43S3, Cm^r^	[26]
pLacZ15-P_*yfdX*_	*yfdX* promoter region cloned into pLacZ15	This study
pLacZ15-P_*hdeDB*_	*hdeDB* promoter region cloned into pLacZ15	This study
pLacZ15-P_*hdeB1*_	*hdeB1* promoter region cloned into pLacZ15	This study
pLacZ15-P_*kvhAS*_	*kvhAS* promoter region cloned into pLacZ15	This study
pLacZ15-P_*hdeD1B2*_	*hdeD1B2* promoter region cloned into pLacZ15	This study
pLacZ15-P_*hdeA*_	*hdeA* promoter region cloned into pLacZ15	This study

^a^ Cm^r^, chloramphenicol resistance; Sm^r^, streptomycin resistance; Km^r^, kanamycin resistance.

**Table 2:** Oligonucleotide primers used in this study.

Purpose	Primer name	Sequence (5' to 3')
**Gene deletions**		
*rcsB*	rcsB-A(+)	AGAGAGCTCTGCAGCTGCTCATCAACA
	rcsB-A(-)	CCCAAGCTTGCGCATCCTTTTCGCGA
	rcsB-B(+)	CCCAAGCTTATTCCCGCCCTTTACGCA
	rcsB-B(-)	TGCTCTAGAGGGGATCCCGGCGAAA
*rcsC*	rcsC-A(+)	TCTAGACAGCTGGCGGAGGAGGCGG
	rcsC-A(-)	CTCGAGGTGCGTAAAGGGCGGGAATAATGG
	rcsC-B(+)	CTCGAGCTTCAGCTCTTTCATTACATCCGCGG
	rcsC-B(-)	GAATTCCCGATCGTCAACCTGCTGCC
*rcsD*	rcsD-A(+)	TCTAGATATTATGCCACTGCTTACTGATTACCCTTC
	rcsD-A(-)	CTCGAGGTTGACTGAGGTGGCGGCGATATTG
	rcsD-B(+)	CTCGAGCAGCTGGCGGAGGAGGCGG
	rcsD-B(-)	GAATTCGTGCGTAAAGGGCGGGAATAATGG
*rcsF*	rcsF-A(+)	CTCGAGACAGATCGGTAAAGCACGCATAGTATT
	rcsF-A(-)	TCTAGAAAGTCGGCGTTATCGTCGGG
	rcsF-B(+)	CTCGAGCTTAACGTCTCGGCGCAATGA
	rcsF-B(-)	GAATTCTGCCCAGCCTGAAACAAAAAAA
*yfdX*	yfdX-A(+)	AGAAGGCCACCGGGGTCATG
	yfdX-A(-)	CTCGAGAAGCATCACCAAACGCAGCC
	yfdX-B(+)	CTCGAGTGGTGGCAGGCAACTGATACTT
	yfdX-B(-)	AGCAGACCGGCTCCGGACT
*hdeA*	hdeA-A(+)	ATGAATTCGGGGTGCTATGGGTAAC
	hdeA-A(-)	ATGGTACCTCGTGCTGAATGGGA
	hdeA-B(+)	ATGGTACCGTGCGCCGATGG
	hdeA-B(-)	ATTCTAGAGCGTCACTGGGCGGATA
*hdeB*	hdeB-A(+)	CCAGATATCCACGGAAGCCTTGTCGCACT
	hdeB-A(-)	CCACTCGAGAATAACCCCCCCGGCATCAG
	hdeB-B(+)	CCACTCGAGAGCCGCCACGGTCTATACGA
	hdeB-B(-)	ACATCGGCGGCTTCTTTCTG
*hdeB1*	hdeB1-A(+)	CCTGATATCATAAGACGAACCGCCATGCC
	hdeB1-A(-)	CCACTCGAGACCGCGGCGCTACTCATTG
	hdeB1-B(+)	CCACTCGAGAAGCGACCGCGGTAAAACG
	hdeB1-B(-)	TAAAAACAGCAGCTGCGCGC
*hdeB2*	hdeB2-A(+)	GAATTCTTTACCTGTCGGCTGGC
	hdeB2-A(-)	GAGCTCCATGATGTTTTCCTGTTTG
	hdeB2-B(+)	GAGCTCATCCTGCGCAGTTTATTCT
	hdeB2-B(-)	GATATCACTCCCCATTTCGCCAGC
*hdeD*	hdeD-A(+)	CCTGATATCCCATCTACCTGACGGCCGG
	hdeD-A(-)	CCACTCGAGGCACGCTGAGGCTTAAGCCC
	hdeD-B(+)	CCACTCGAGCAGGCATGCCGTTTATATCGAA
	hdeD-B(-)	TGCGCTCTCTCAGGGTGGAA
*hdeD1*	hdeD1-A(+)	TGAATTCTTGCGGTCGCCTGTTTCTT
	hdeD1-A(-)	TTCTAGAGAATATCAATGCCATCGCCACAGA
	hdeD1-B(+)	TTCTAGAATCCTGCGCAGTTTATTCTTTTCTGC
	hdeD1-B(-)	TGATATCGCAGTAAACCAGAAGTGTCCAGAAGGT
**Point mutation**		
*hdeA* F44A	hdeA F44A(+)	CTGCCGTAGGCTGAGCATCTTCATTTACC
	hdeA F44A(-)	GGTAAATGAAGATGCTCAGCCTACGGCAG
*rcsB* D56A	rcsB D56A(+)	ATGTGCTGATCACCGCTCTGTCCATG
	rcsB D56A(-)	ATGGACAGAGCGGTGATCAGCACATG
*rcsB* D56E	rcsB D56E(+)	ATGTGCTGATCACCGAGCTGTCCATG
	rcsB D56E(-)	ATGGACAGCTCGGTGATCAGCACATG
**Gene expression**		
pRK415-rcsB	pRK415 rcsB(+)	CCCGGATCCAACTGCGGGTCAACTTT
	pRK415 rcsB(-)	CCCGGATCCTTGTCTGTCCAAGCCGGTCA
pRK415-*yfdX*	pRK415 yfdX(+)	GAAGGATCCCAGCAATACCGCCATCAGG
	pRK415 yfdX(-)	GAATTCTGCGCTCTCTCAGGGTGGAAC
pRK415-*hdeA*	pRK415 hdeA(+)	TGGATCCGAATAGCTTAACTCTATCGTAAATCGC
	pRK415 hdeA(-)	TGGTACCATTGTGGCATTCCCCTGG
pRK415-*hdeB*	pRK415 hdeB(+)	AGAAGCTTATGGCGGTATTGCTGTTTATC
	pRK415 hdeB(-)	ATGGATCCTTATTTTTTGATGACCGCGC
pRK415-*hdeD*	pRK415 hdeD(+)	ACAAGCTTCAAACGCAGCCAGCTTAAAAAATATC
	pRK415 hdeD(-)	ATGGATCCTTAAGCCTCAGCGTGCTTC
pRK415-*hdeDB*	pRK415 hdeD(+)	ACAAGCTTCAAACGCAGCCAGCTTAAAAAATATC
	pRK415 hdeB(-)	ATGGATCCTTATTTTTTGATGACCGCGC
pET30a-*yfdX*	pET30a yfdX(+)	GAATTCACAGATAGCGCGACGGCAGCGCCAG
	pET30a yfdX(-)	CTCGAGTGCGCTCTCTCAGGGTGGAAC
**EMSA**		
*yfdX*	yfdX(+)-BIOTIN	Biotin-GGATCCGCTATCTGTTGCCCATACCGGA
	yfdX(+)	GGATCCGCTATCTGTTGCCCATACCGGA
	yfdX(-)	AGATCTAATTGCTCCGCAGATCCCGGT
*hdeB1*	hdeB1(+)	GACGGATCCGATTATCGCATTCATGGGGGC
	hdeB1(-)-BIOTIN	Biotin-AGATCTCAGATGTTTCCAAACCCATTTTC
	hdeB1(-)	AGATCTCAGATGTTTCCAAACCCATTTTC
**Promoter assay**		
P-*yfdX*	lacZ-YfdX(+)	GGATCCGCTATCTGTTGCCCATACCGGA
	lacZ-YfdX(-)	AGATCTAATTGCTCCGCAGATCCCGGA
P-*hdeDB*	lacZ-HdeDB(+)	GAAGGATCCCAGCAATACCGCCATCAGG
	lacZ-HdeDB(-)	CCTAGATCTATCACCAAACGCAGCCAGC
P-*hdeB1*	lacZ-HdeB1(+)	GACGGATCCGATTATCGCATTCATGGGGGC
	lacZ-HdeB1(-)	CCACCGCGGCGCTACTCATT
P-*kvhAS*	lacZ-KvhAS(+)	GACGGATCCGATTATCGCATTCATGGGGGC
	lacZ-KvhAS(-)	CCCAGATCTCCGAGAACTCACCTTAATAAGAGCA
P-*hdeD1B2*	lacZ-HdeD1B2(+)	TGGATCCTTAATGCTTGTCATCTATCAGGCC
	lacZ-HdeD1B2(-)	TAGATCTATGAATATCAATGCCATCGCCACAGA
P-*hdeA*	lacZ-HdeA(+)	TGGATCCGAATAGCTTAACTCTATCGTAAATCGC
	lacZ-HdeA(-)	TAGATCTCCGATGGCACCAAAAACCAATG

### Construction of the gene deletion mutants and the gene complement strains

Specific gene deletion was introduced to the chromosome of *K*. *pneumoniae* CG43S3 by using an allelic-exchange strategy essentially as described [40]. In brief, the DNA fragments of 1 kb flanking both ends of *rcsB*, *rcsC*, *rcsD*, *rcsF*, *yfdX*, *hdeDB*, *hdeB1*, *hdeA* and *hdeD1B2* gene were amplified using PCR with the primer sets in Table 2. The two amplified DNA fragments were cloned into the suicide vector pKAS46 [45]. The resulting plasmid was transformed into *E*. *coli* S17-1λ*pir* and then mobilized by conjugation to the streptomycin-resistant strain, *K*. *pneumoniae* CG43S3. Several kanamycin-resistant transconjugants, with the plasmid integrated into the chromosome through homologous recombination, were selected from M9 agar plates supplemented with kanamycin and propagated in 2 ml of LB broth overnight. A small aliquot of the culture was then plated on LB agar containing 500 μg/ml of streptomycin. Lastly, the streptomycin-resistant and kanamycin-sensitive colonies were isolated, and the specific gene deletion of *rcsB*, *rcsC*, *rcsD*, *rcsF*, *yfdX*, *hdeDB*, *hdeB1*, *hdeA* and *hdeD1B2* were verified with PCR analysis. For complementation analysis, the DNA region containing *rcsB*, *yfdX*, *hdeB*, *hdeD*, *hdeDB*, and *hdeA* were amplified using PCR with respective primer pairs in Table 2, and the DNA fragments cloned into pRK415, and transferred to the specific gene deletion mutant by conjugation.

### Site-directed mutagenesis

The site-directed mutation plasmids pRK415-*hdeA*-F44A, pRK415-*rcsB*-D56A and pRK415-*rcsB*-D56E were generated using PCR-based mutagenesis with the plasmid pyT&A-*hdeA* and pyT&A-*rcsB* as template to substitute the phenylalanine at residue 44 of HdeA with alanine and aspartic acid at residue 56 of RcsB with glutamic acid or alanine. pyT&A-*rcsB* and pyT&A-*hdeA* were amplified with the point mutation primer sets *rcsB*-D56E(+)/*rcsB*-D56E(−), *rcsB*-D56A(+)/*rcsB*-D56A(−) and *hdeA*-F44A(+)/*hdeA*-F44A(-) encompassing the mutation site by using PfuUltra II Fusion HS DNA polymerase (Agilent Technologies) to generate mutant alleles of *rcsB* and *hdeA*, respectively. The PCR product was resolved on an agarose gel, recovered, treated with DpnI to remove the template plasmid and transformed into *E*. *coli* JM109. The point mutation allele of the recombinant plasmid was later confirmed by DNA sequencing. The mutated fragment *rcsB*-D56E, *rcsB*-D56A and *hdeA*-F44A were subcloned into plasmid pRK415 to yield pRK415-rcsB-D56E, pRK415-rcsB-D56A and pRK415-hdeA-F44A, respectively. The site-directed mutation plasmids were then individually mobilized from *E*. *coli* S17-1 λ*pir* to the *K*. *pneumoniae* CG43S3 strain by conjugation.

### Acid stress survival assessment

Overnight-grown bacteria diluted 1:20 in LB broth were incubated at 37°C to OD_600_ of 0.6~0.7 (exponential phase) or 1.0~1.1 (stationary phase). An aliquot of the bacteria was collected by centrifugation, resuspended in the acidic LB broth (adjusted to pH 4.4 by HCl) and subjected to 37°C incubation under shaking cultured condition (130 rpm) for 1 h adaptation before the acid challenge. After adaptation, the bacteria (approximately 5x10^8^~1x10^9^ CFU/ml) were transferred to the acidic M9 medium (adjusted to pH 2.5 by HCl) and incubated at 37°C for 30 min under shaking cultured condition (130 rpm). After the acid stress treatment, the bacteria were diluted serially to 10^−6^ and 10 μl of each sample was spotted onto LB agar plate and incubated at 37°C overnight. The presented results are representative of at least three independent experiments. The survival was calculated by dividing the number of colonies after acid treatment by that before the treatment (after pH4.4 acid adaptation). Each sample was assayed in triplicate, and at least three independent experiments were conducted. The data were calculated from three independent experiments and are shown as the mean and standard deviation from that samples. Student’s *t*-test was used to determine differences between groups and values of P<0.05 and P<0.01 were considered statistically significant difference.

### 2D-PAGE analysis

Overnight-grown bacteria diluted 1:20 in LB broth were incubated at 37°C to OD600 of 0.6~0.7. Since cells died profoundly upon pH 2.5 treatment, the bacteria were transferred to the acidic LB broth (pH 4.4) and the incubation continued for 1 h before protein collection. Bacteria were finally collected by centrifugation at 3000 rpm for 30 min, washed three times with wash buffer (10 mM Tris-HCl pH 7.5, 250 mM sucrose), resuspended in 3 ml lysis buffer (10 mM Tris-HCl pH 7.5). The bacteria were lysed by sonication followed by centrifugation at 3000 rpm for 40 min. The supernatants were treated by DNase (20 units) and RNase (20 units) at 37°C for 45 min, followed by centrifugation at 15,000 rpm for 30 min to remove the insoluble portions. Finally, the supernatants were passed through the 10 kDa microcon (Millipore), and the filtrates were freeze-dried and stored at -80°C before use. Aliquot of sample (250 μg) was dissolved in 250 μl rehydration buffer (2 M thiourea, 7 M urea, 2% 3-[(3-Cholamidopropyl) dimethylammonio]-1-propanesulfonate (CHAPS), 1% immobilized pH gradient (IPG) buffer, 0.002% bromophenol blue, 0.28% Dithiothreitol (DTT)) followed by centrifugation at 15,000 rpm for 20 min. Each sample was added into holder, and then the strip (pH 4–7, 13 cm) was placed and an appropriate amount of dedicated mineral oil was added. The holder was inserted in IPGphor (GE Healthcare) to execute isoelectric focusing. After finishing isoelectric focusing, the strip was soaked into the equilibration buffer (50 mM Tris-HCl pH 8.8, 6 M urea, 30% glycerol, 2% SDS, 0.002% bromophenol blue) containing 1% DTT for 15 min and then soaked into the equilibration buffer containing 2.5% idoacetamide (IAA) for 15 min. After the pretreatment of strip, 12.5% polyacrylamide gel was used to run 2D-PAGE. Finally, Sypro Ruby (Invitrogen) and Typhoon 9200 (GE Healthcare) were respectively used to stain and scan the gel, and Image Master 2D platinum 6.0 (GE Healthcare) was used to detect and quantify the protein spots. Student’s *t*-test was used to determine relative volume of the protein spots between CG43S3 and CG43S3Δ*rcsB*. The protein spots with more than 1.5 fold differences of the relative volume between the two proteomes were isolated and subjected to mass spectrometry analysis.

### Mass spectrometry analysis

The gel digestion method was modified from the previous research [47]. Protein spots were excised from the gel and washed using wash buffer (50% acetonitrile, 25 mM NH_4_HCO_3_) for 15 min. After removing the wash buffer, the gel pieces were shrunk by dehydration in 100% acetonitrile, swelled by rehydration in NH_4_HCO_3_ (100 mM) for 5 min, and shrunk again by addition of 100% acetonitrile. The liquid phase was removed, and the gel pieces were completely dried at room temperature. Then, 2 μl of the digestion buffer (25 mM NH_4_HCO_3_, 20 ng/μl trypsin (Promega) was added and the gel pieces were placed at 4°C for 1 h. Then, a sufficient volume of NH_4_HCO_3_ (25 mM) was added to keep the gel pieces wet, and the gel pieces were placed at 37°C overnight for enzymatic cleavage. Peptides were extracted by addition of 2 μl of the buffer containing 100% acetonitrile and 1% trifluoroacetic acid and sonication of the gel pieces for 10 min. The extraction step was repeated three times. Then, the collected samples were subjected to MALDI-TOF analysis by Academia Sinica Proteomic mass spectrometry common facility (http://www.ibc.sinica.edu.tw/Facility_Mass_E.asp).

### Chaperone assay

As described in ref [20, 48], the chaperone assay used alcohol dehydrogenase (ADH) as the target substrate for chaperone protein. Initially, ADH (10 mM) was mixed with different concentrations of the purified recombinant YfdX protein. Then, each of the mixtures was treated with acidic distilled water (adjusted to pH 1.0 with HCl) for 15 min, followed by centrifugation at 15,000 rpm for 10 min to remove the insoluble portions. Finally, the supernatants were analyzed by SDS-PAGE.

### Measurement of promoter activity

The putative promoter region of *kvhAS*, *yfdX*, *hdeDB*, *hdeB1*, *hdeA*, and *hdeD1B2* were PCR-amplified using primers sets in Table 2. The amplicons were then cloned into placZ15 [25] to generate P_*yfdX*_-lacZ, P_*hdeDB*_-lacZ, P_*hdeB1*_-lacZ, P_*kvhAS*_ -lacZ, P_*hdeA*_-lacZ, and P_*hdeD1B2*_-lacZ. The promoter-reporter plasmids were individually mobilized into *K*. *pneumoniae* CG43S3Δ*lacZ* strains through conjugation from *E*. *coli* S17-1 λ*pir*. The β-galactosidase activity was measured for the bacteria grown to exponential phase with OD_600_ of 0.6~0.7 [26]. The promoter activity was expressed as Miller units. Each sample was assayed in triplicate, and at least three independent experiments were conducted. The data were calculated from three independent experiments and are shown as the mean and standard deviation from that samples. Student’s *t*-test was used to determine differences between groups and values of P<0.05 and P<0.01 were considered statistically significant difference.

### RcsB expression plasmid construction

The coding regions of *rcsB* was PCR-amplified with the primer pairs listed in Table 2. The amplified DNA was cloned into cloning vector yT&A (Yeastern Biotech Co., Ltd.), and the resulting recombinant plasmids named pyT&A-*rcsB*. For protein expression and purification, the coding regions from pyT&A-*rcsB* was subcloned into pET30a (Novagen) and resulting the plasmids pET30a-*rcsB*.

### Expression and purification of the recombinant proteins

The plasmids pET30a-*rcsB* was individually transformed into *E*. *coli* NovablueDE3, and protein production was induced with 0.5 mM isopropyl-β-D-thiogalactopyranoside (IPTG) for 5 h at 37°C. The overexpressed protein was then purified from the soluble fraction of the cell lysate by affinity chromatography using His-Bind resin essentially according to the QIAexpress expression system protocol (Qiagen). The purified RcsB protein was dialyzed against Tris-buffered saline (pH 7.4) containing 10% glycerol at 4°C overnight, followed by condensation with PEG 20000. The protein purity was determined using SDS-PAGE.

### DNA electrophoretic mobility shift assay (EMSA)

The putative promoter region of *yfdX* and *hdeB1* were PCR-amplified using biotin-labeled primer pairs *yfdX*(+)-BIOTIN/*yfdX*(-) and *hdeB1*(-)-BIOTIN/*hdeB1*(+), and non-labeled primer pairs *yfdX*(+)/(-), and *hdeB1*(+)/(-). The DNA binding reaction was performed in a 20 μl interaction buffer and the mixture resolved using 5% native polyacrylamide gel electrophoresis. The interaction buffer, for RcsB and each of the above-mentioned promoters, contained 0.5 mM MgCl_2_, 0.1% Nonidet P-40, 0.05 mg/ml BSA, 50 ng/μl of sheared salmon sperm DNA and 5% glycerol [39]. After being transferred onto a Biodyne B Nylon membrane, the biotin-labeled DNA was detected using a LightShift chemiluminescent EMSA kit (Pierce).

### YfdX antisera preparation

The y*fdX* coding sequence was amplified using PCR from *K*. *pneumoniae* CG43S3 genome, and ligated into expression vector pET30a (Novagen). The plasmid pET30a-*yfdX* was transformed into *E*. *coli* JM109, and the gene expression of the recombinant protein His_6_-YfdX was induced with 0.5 mM IPTG for 5 h at 37°C. The soluble His_6_-YfdX protein was purified using a nickel column (Novagen, Madison, WI, USA). Then Then the polyclonal YfdX antisera was prepared by LTK BioLaboratories (Taoyuan, Taiwan, ROC). The procedure was as follows: 1 mg purified protein emulsified with 500 μl complete Freund’s adjuvant was used to immunize New Zealand white rabbits weighing 2.0~2.5 kg by intramuscular injection. The rabbits were boosted three times at 2 week intervals with 500 μg purified YfdX recombinant protein. The YfdX antisera was obtained by intracardiac puncture 8 weeks later.

### Western blot analysis

Aliquots of the total cellular lysates were resolved through sodium dodecyl sulfate polyacrylamide gel electrophoresis, and the proteins were electrophoretically transferred onto a polyvinylidene difluoride (PVDF) membrane (Millipore, Billerica, MA, USA). After incubation with 5% skim milk at room temperature for 1 h, the membranes were washed 3 times using phosphate buffered saline with 0.1% Tween 20 (PBST), and were then incubated with an anti-GAPDH (1:5000 dilution) (GeneTex, GTX100118) or anti-YfdX (1:10000 dilution) antiserum at room temperature for 2 h. Again, the membranes were washed 3 times with 1X PBST, and subjected to incubation with a 1:5000 dilution of the secondary antibody, alkaline phosphatase-conjugated anti-rabbit immunoglobulin G (Millipore, AP132A), at room temperature for 1 h. Finally, the blots were rewashed, and the secondary antibodies bound on the PVDF membrane were detected using chromogenic reagents 5-bromo-4-chloro-3-indolyl phosphate (BCIP) and nitro blue tetrazolium (NBT). Data quantification was done using ImageJ 1.46r (http://imagej.nih.gov/ij/).

## Results

### Deletion of *rcsB* or *rcsD* reduced acid survival

**Fig 2:**
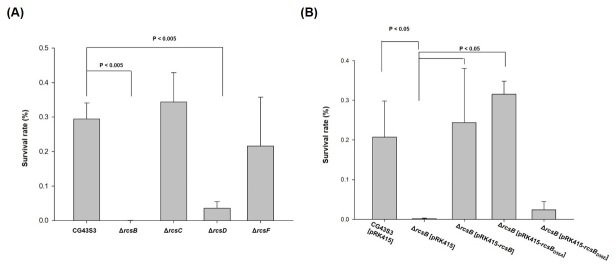
Acid survival analysis of the gene deletion effect of *rcsB*, *rcsC*, *rcsD*, and *rcsF*. Acid survivals of CG43S3, Δ*rcsB*, Δ*rcsC*, Δ*rcsD*, and Δ*rcsF* (A), and CG43S3 [pRK415], Δ*rcsB*[pRK415], Δ*rcsB*[pRK415-*rcsB*], Δ*rcsB*[pRK415-*rcsB*_D56A_], and Δ*rcsB*[pRK415-*rcsB*_D56E_] (B) are shown. Acid survival was determined essentially as following. The mutant and complement strains were grown to the exponential phase (OD_600_ 0.6~0.7) and then an aliquot of bacteria was treated with acid stress. Error bars indicate standard deviations of three independent experiments done in triplicate.

To analyze the deletion effect of *rcsB* on bacterial AR and whether other Rcs components were also involved in the RcsB-dependent acid stress response, we constructed *rcsB*, *rcsC*, *rcsD*, and *rcsF* deletion mutant strains and subjected them to acid treatment modified from previous studies [49, 50]. The bacterial strains were cultured at a pH of 4.4 for 1 h for acid adaptation and then at a pH of 2.5 for 30 min for acid stress treatment. The survival of bacterial strains that were subjected to the pH 4.4 treatment were first analyzed to reveal that the growth of each bacterial strains was at the comparable levels. As shown on the left panel of S2 Fig, the acid adaptation (pH 4.4 for 1h) had no apparent influence on the survival of each bacterial strain. The acid survival was quantitatively and qualitatively determined to assess the deletion effects. As illustrated in Fig 2A and S2A Fig, after treatment at pH 2.5, the acid stress resistance was reduced when *rcsB* or *rcsD* was removed. By contrast, the survival of the mutants Δ*rcsC* and Δ*rcsF* was similar to that of the parental strain.

### AR may be affected by the phosphorylated form of RcsB

During bacterial signal transduction, RcsD kinase receives the phosphate group from RcsC and subsequently relays it to RcsB. Because the removal of *rcsD* as well as *rcsB* deletion reduced the AR, we assumed that the relay of the phosphoryl group was blocked, thereby affecting the phosphorylated form of RcsB. To investigate whether the phosphorylation status of RcsB influences the acid stress resistance, site-directed mutants, RcsB_D56A_ and RcsB_D56E_, which mimic the nonphosphorylated and phosphorylated forms of RcsB, respectively, were created. As illustrated in Fig 2B and S2B Fig, the deletion effect was complemented by introducing an RcsB-expression plasmid pRK415-*rcsB* into the mutant, indicating an involvement of RcsB in the regulation of the acid stress response. In addition, the acid stress sensitivity of Δ*rcsB* was also significantly reduced by increasing the expression of RcsB_D56A_. By contrast, the deficiency of Δ*rcsB* could not be rescued through the expression of RcsB_D56E_. These findings suggest that RcsB in the nonphosphorylated form activates the AR response. Notably, we also could not rule out the possibility that the point mutation influenced the protein stability or conformation of RcsB_D56E_ and resulted in the loss of the regulation function. More experiments are warranted to support this finding.

### Deletion of *rcsB* blocked YfdX production

**Fig 3:**
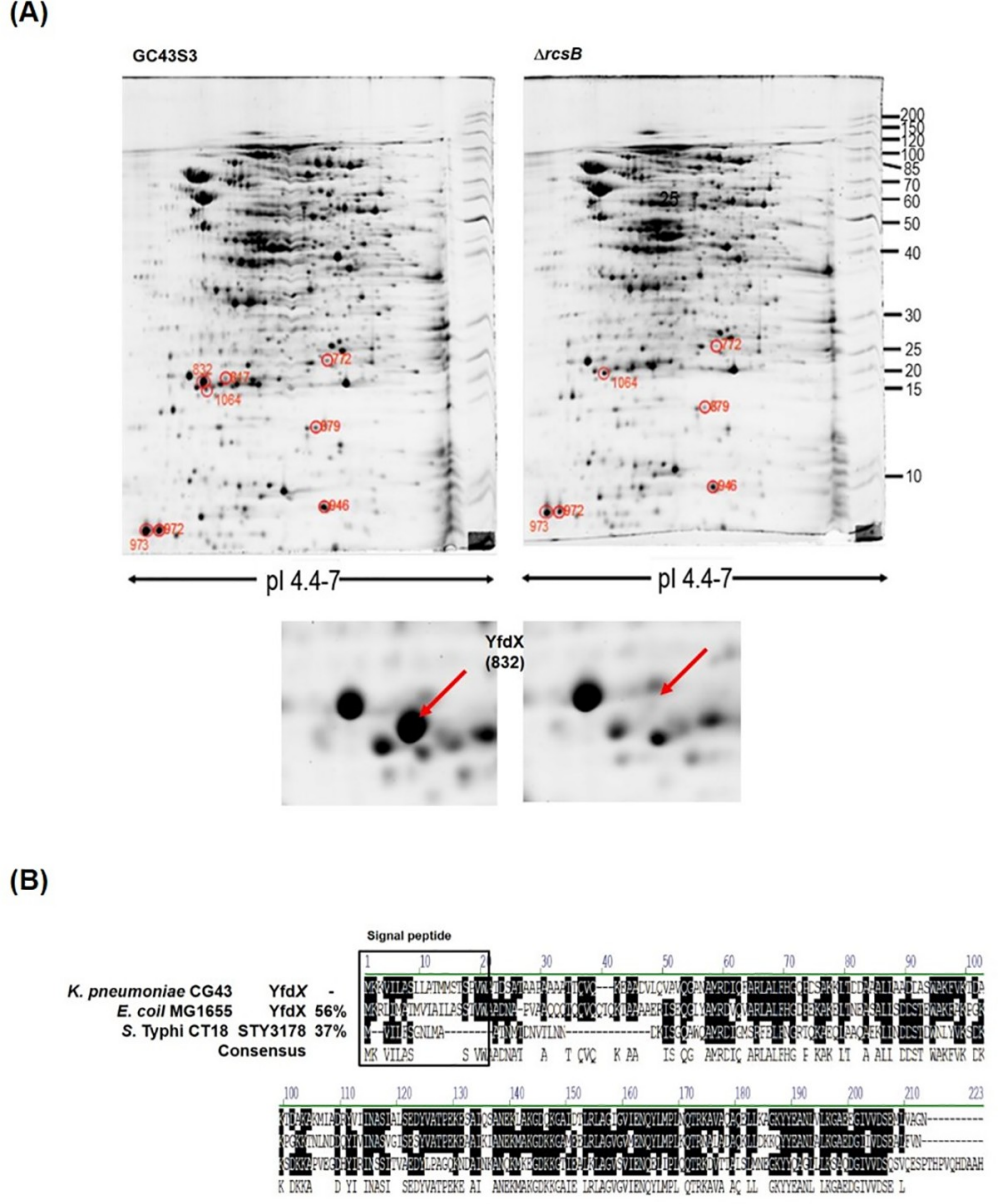
Proteome analysis of the *rcsB* deletion effects. (A) Comparative proteome analysis of *K*. *pneumoniae* CG43S3 and CG43S3Δ*rcsB*. Representative SYPRO Ruby-stained gels derived from CG43S3 (WT) and Δ*rcsB* are shown. The exponential phase bacteria were incubated in LB broth at pH 4.4 for 1 h. proteome analysis was then performed. Spot 832, present only in CG43S3, was isolated and identified as YfdX through mass spectrometry. (B) Sequence comparison of YfdX of *K*. *pneumoniae* CG43S3, *S*. Typhi CT18, and *E*. *coli* MG1655. The predicted signal peptide (according to the SignalP 4.1 server) are marked.

Either pH 4.4 or pH 5 treatment had no apparent effect on the transcriptional levels of *rcsB*, indicating that the *rcsB* expression was not acid induced (S3 Fig). Moreover, no AR2 system or conserved AFI was identified in the *K*. *pneumoniae* genome. To elucidate the RcsB-mediated regulation of the response to acid stress, a comparative proteome analysis of CG43S3 and CG43S3Δ*rcsB* was performed. As depicted in Fig 3A, after incubation at pH 4.4 for 1 h for acid adaptation, the spots marked 772, 817, 832, 879, 946, 972, 973, and 1064 exhibited differences between the proteomes of the mutant strain CG43S3Δ*rcsB* and the parental strain CG43S3. As shown in S1 Table, *rcsB* deletion led to 2.18-, 1.90-, and 1.52-fold decreased expression of spots 772, 972, and 973, respectively. Spots 817 and 832 were present only in CG43S3. Spot 832, which exhibited obviously different, was isolated, analyzed through mass spectrometry, and identified as YfdX. Unlike the gene in *E*. *coli*, *yfdX* is located next to *hdeDB* in *K*. *pneumoniae* CG43 (Fig 1). Sequence comparison (using Vector NTI 10.3) revealed that the YfdX of *K*. *pneumoniae* had a similarity of 56% and 37% to *E*. *coli* MG1655 and *S*. Typhi CT18, respectively, and a signal peptide could be predicted for all three YfdX sequences (Fig 3B). The YfdX of *S*. Typhi CT18, STY3178, may play a role in multidrug resistance, and the F67, Y109, Y186, and Y187 residues for STY3178’s interaction with antibiotics [51] are conserved in *K*. *pneumoniae* YfdX (Fig 3B).

### Deletion of *yfdX*, *hdeD*, and *hdeB1* reduced acid survival

**Fig 4:**
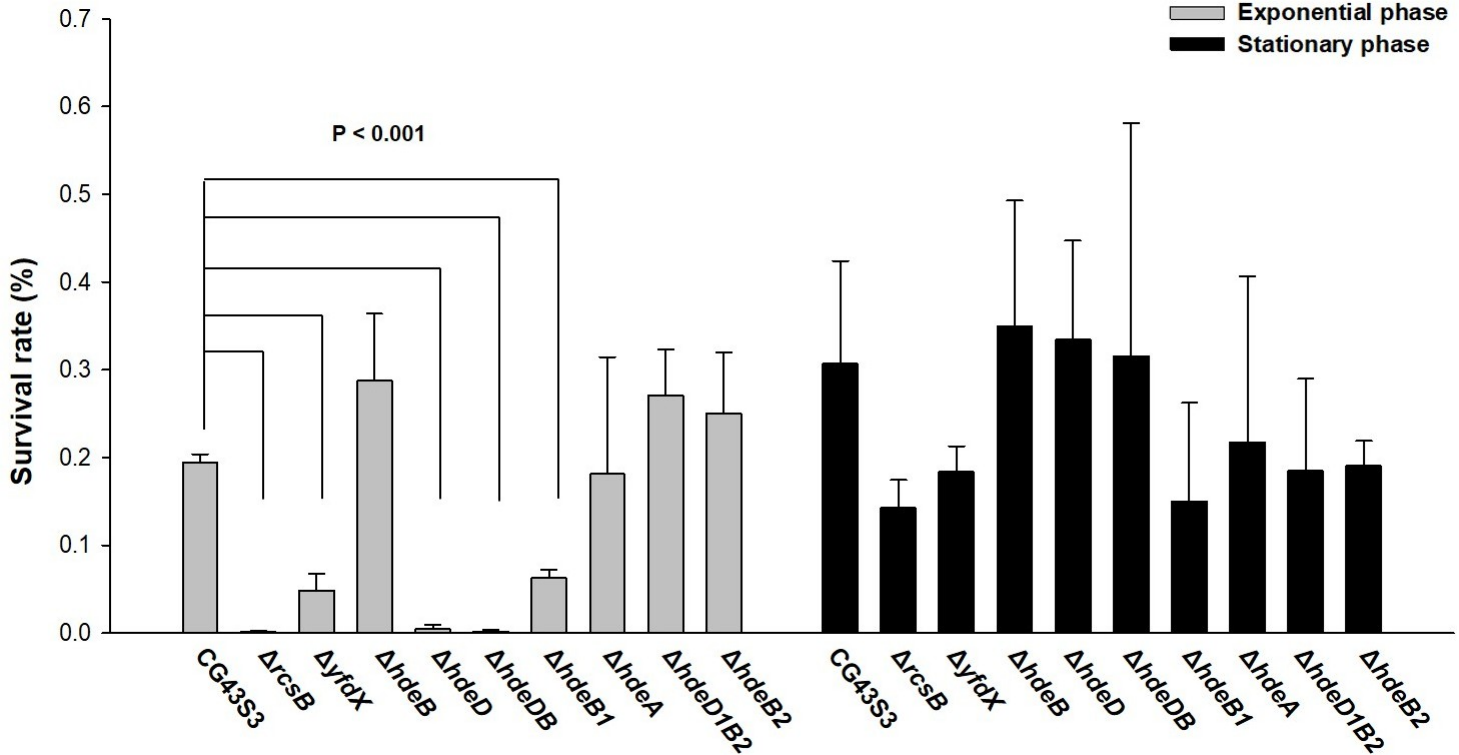
Effects of *yfdX* and *hde* genes deletions on acid survival. The *hde* genes include *hdeB*, *hdeD*, *hdeDB*, *hdeB1*, *hdeA*, *hdeB2*, and *hdeD1B2*. The mutant strains were grown to the exponential phase (OD_600_ 0.6~0.7) or stationary phase (OD_600_ 1.0~1.1) and treated with acid stress. The relative survival was determined as the ratio of viable counts relative to the inoculum before acid stress treatment. Error bars indicate standard deviations of three independent experiments done in triplicate.

As shown in Fig 1, the clustered location of *yfdX* with the chaperone encoding gene *hdeDB* and the *evgAS* orthologous gene *kvhAS* suggested a similar functional role. To determine the involvement of YfdX and Hde proteins in the acid stress response, the specific gene-deletion mutants were constructed, and the effects of deletion on bacterial susceptibility to acid stress were analyzed. In *E*. *coli*, the expression of *hdeA* is repressed by a histone-like nucleoid structuring protein (H-NS) and is activated when the bacteria enter the stationary phase [34, 52, 53]. Therefore, the deletion effects of bacteria grown at the exponential and stationary phases were measured. Each gene deletion was confirmed, showing no apparent effect on the bacterial growth before pH 2.5 treatment (the left panel of S4 Fig). As shown in Fig 4 and S4 Fig, the deletion of *yfdX*, *hdeD*, and *hdeB1* reduced the survival after acid treatment, whereas the deletion of *hdeA*, *hdeB*, *hdeB2*, or *hdeD1B2* had no apparent effect on the acid survival at the exponential phase. However, no significant difference in acid survival was observed among the mutant strains at the stationary phase.

### Overexpression of HdeA enhanced the AR

**Fig 5:**
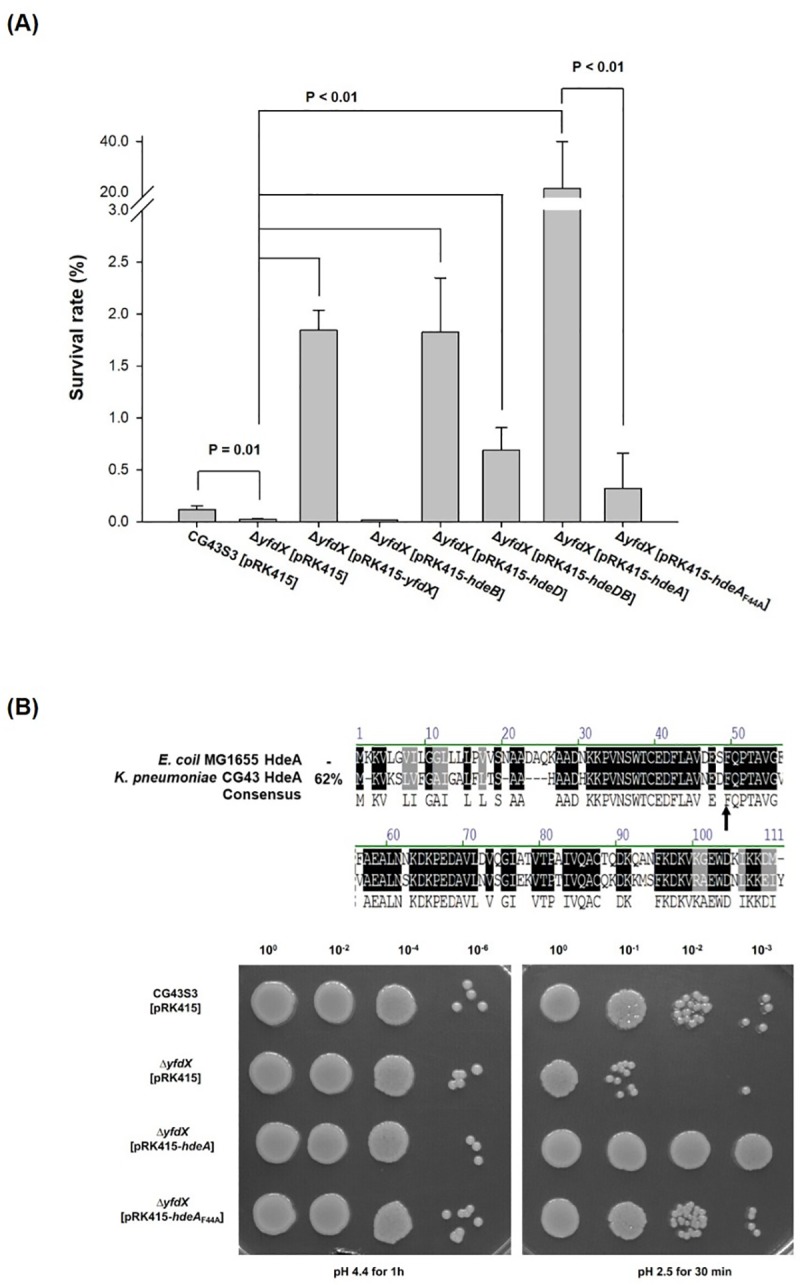
YfdX may function as a chaperone protein. (A) Complementation analysis by transforming Δ*yfdX* with the plasmid pRK415 carrying gene encoding YfdX, HdeB, HdeD, HdeDB, HdeA, or HdeA_F44A_. The complement strains were grown to the exponential phase and treated with acid stress. The relative survival was determined as the ratio of viable counts relative to the inoculum before acid stress treatment. Error bars indicate standard deviations of three independent experiments done in triplicate. (B) Upper panel, sequence comparison of HdeA of *K*. *pneumoniae* CG43S3 and *E*. *coli* MG1655. The sequence comparison of HdeA family proteins between *E*. *coli* MG1655 and *K*. *pneumoniae* CG43S3 are shown. The conserved critical residue (F44) for HdeA chaperone activity is indicated by an arrow. Lower panel, complementation effects of HdeA and HdeA_F44A_, the critical residue mutation protein on Δ*yfdX* strain. The complement strains were grown to the exponential phase and treated with acid stress and the acid survival analysis was performed.

In *E*. *coli*, the periplasmic chaperone activity of HdeA, HdeB, and HdeD has been demonstrated [17, 18, 20, 54, 55]. A recent report demonstrated that *Salmonella* YfdX also exhibited a periplasmic chaperone activity [56]. To investigate whether the *yfdX* deletion effect could be complemented by the chaperone activity, the plasmid pRK415 carrying *hdeA*, *hdeB*, *hdeD*, or *hdeDB* was individually used to transform the Δ*yfdX* mutant, and then the acid survivals were determined. As illustrated in Fig 5A and S5A Fig, introducing the *yfdX* expression plasmid pRK415-*yfdX* into the Δ*yfdX* mutant restored the bacterial survival. Compared with the survival of Δ*yfdX* [pRK415-*yfdX*], Δ*yfdX* [pRK415-*hdeD*] and Δ*yfdX* [pRK415-*hdeDB*] exerted similar survival levels, and notably, Δ*yfdX* [pRK415-*hdeA*] exhibited a higher level of survival after pH 2.5 treatment. A comparison of sequences, as depicted in the upper panel of Fig 5B, indicated that *K*. *pneumoniae* HdeA exhibits 62% sequence identity with *E*. *coli* HdeA, and the critical residue for chaperone activity is conserved [57]. To further confirm that the chaperone activity of *K*. *pneumoniae* HdeA may compensate for the *yfdX* deletion effect, the critical residue phenylalanine 44 of HdeA was selected for site-directed mutation. The plasmid pRK415 carrying *yfdX*, *hdeA*, or *hdeA*_*F44A*_ was individually used to transform the Δ*yfdX* mutant, and then acid survival rates were determined. Again, no differences in growth were confirmed in each bacterial strain before the pH 2.5 treatment. As illustrated in Fig 5A and the lower panel of Fig 5B, the deletion effects of *yfdX* could be entirely complemented by increasing the expression of YfdX, HdeA, and HdeA_F44A_. This finding suggests that YfdX as well as HdeA functions as a periplasmic chaperone. The acid survival of Δ*yfdX*[pRK415-HdeA_F44A_] was less than that of Δ*yfdX*[pRK415-HdeA] indicating that the phenylalanine is crucial for determining HdeA chaperone activity.

**Fig 6:**
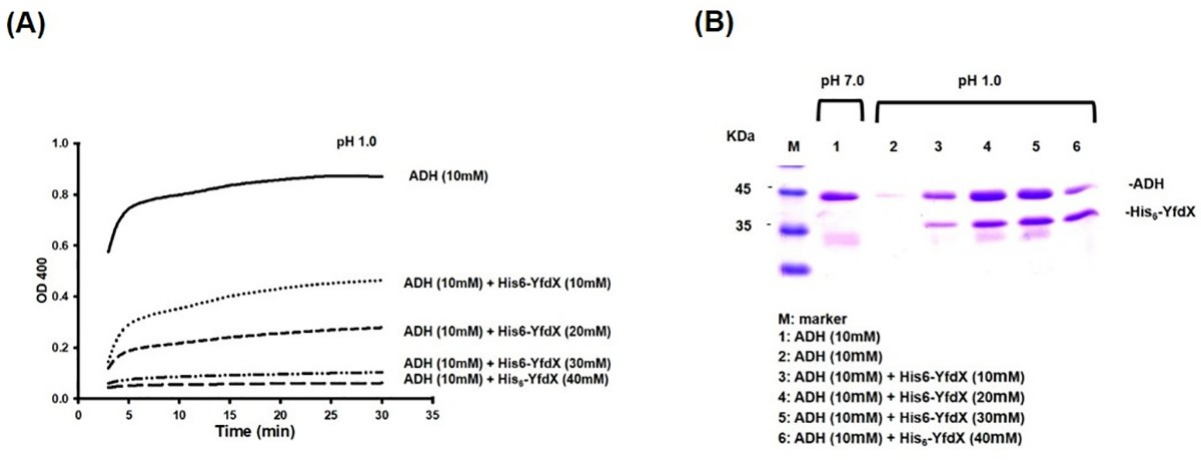
The recombinant YfdX exhibits a chaperone activity. (A) Samples containing ADH and the recombinant His_6_-YfdX were subjected to pH 1.0 treatment and then their OD_400_ were measured. The decreasing optical density corresponds to more aggregated protein formed in the solution. (B) Samples containing ADH and the recombinant His_6_-YfdX were subjected to treatment for 15 min at pH 1.0 or 7.0, and the insoluble aggregated proteins were removed. The supernatant fractions containing soluble protein were analyzed by SDS-PAGE.

Although the crystal structure of *K*. *pneumoniae* YfdX has been resolved (PDB 3DZA), its functional role in the bacteria remains unknown. As shown in Fig 6, alcohol dehydrogenase, a commonly used substrate protein for chaperone activity analysis, was insoluble and became aggregated when the protein incubation switched from pH 7 to 1. The acid-induced aggregation significantly decreased after the addition of the purified recombinant YfdX, as assessed and visualized using optical density measurement and SDS-PAGE, respectively. This supports the possibility that YfdX proteins function as periplasmic chaperones to protect bacteria from acid stress damage.

### RcsB regulates *yfdX*, *hdeDB*, and *hdeB1* expression at the transcriptional level

**Fig 7:**
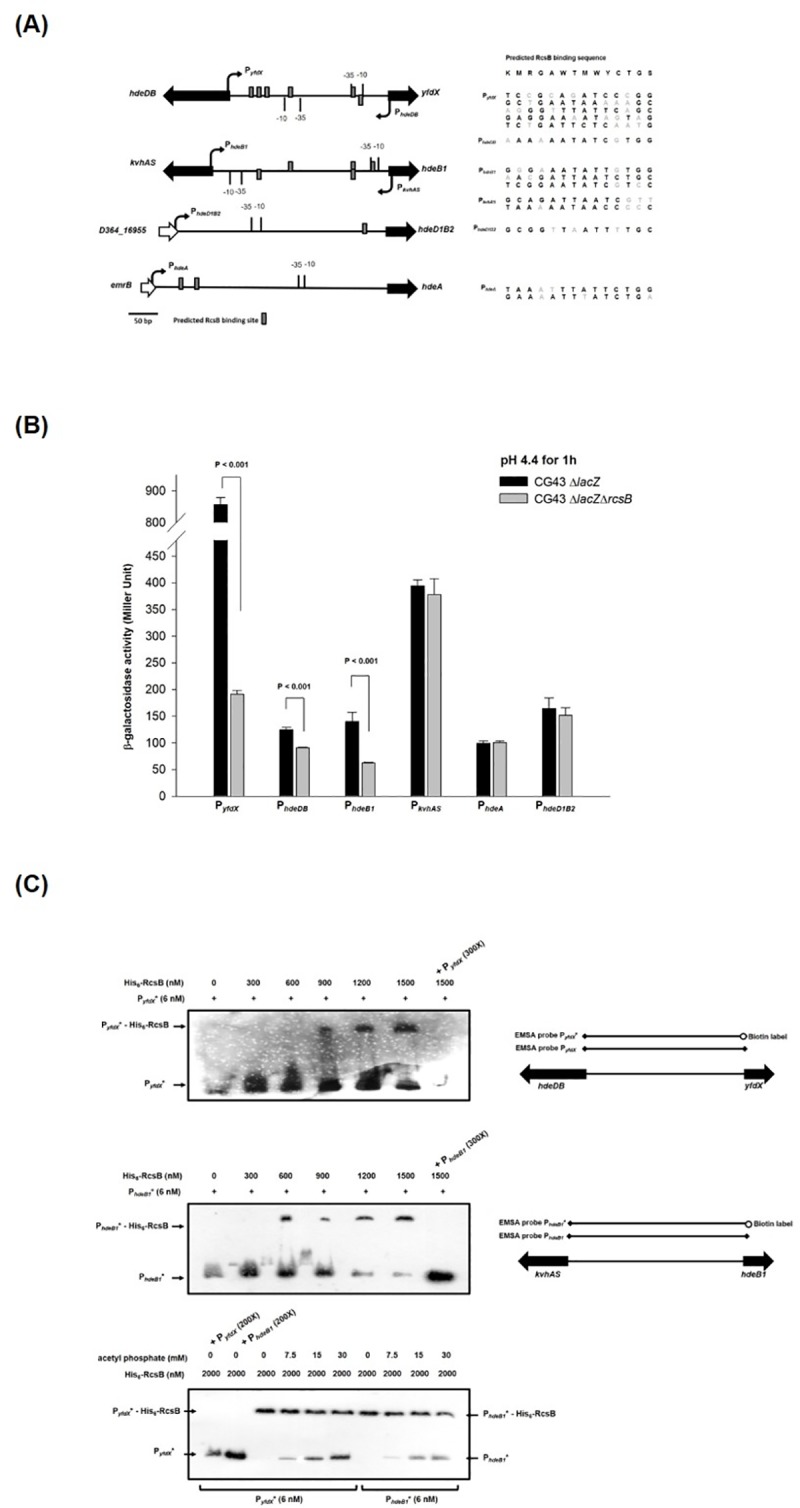
RcsB regulation on the expression of *kvhAS*, *yfdX*, and *hde* genes. (A) The promoter regions of *yfdX* and *hde* genes, and predicted RcsB binding sites (KMRGAWTMWYCTGS, W = A or T, K = G or T, M = A or C, R = A or G, Y = C or T and S = C or G) are marked. (B) The promoter activity was assessed by monitoring the expression of β-galactosidase on the plasmid pLacZ15 cloned with the promoter regions of target genes on Δ*lacZ* and Δ*lacZ*Δ*rcsB* strains, respectively. Bacteria grown to the exponential phase were resuspended in the acidic LB broth (pH 4.4) for 1 h for acid adaptation and then measured the promoter activity. Error bars indicate standard deviations of three independent experiments done in triplicate. (C) EMSA for the interaction between the recombinant RcsB and the putative promoter of *yfdX* or *hdeB1*. The different reaction mixtures of the recombinant His_6_-RcsB and the biotin-labeled probe P_yfdX_* or P_hdeB1_* were resolved on the polyacrylamide gel, and the binding specificity was investigated by adding nonlabeled probe *P*_*yfdX*_ or P_*hdeB1*_ at a 300-fold concentration. To determine the phosphorylation effect in the interaction, different concentrations of acetyl phosphate was added in the reaction buffer.

To determine whether RcsB regulates the expression of *yfdX* and *hde*, the putative promoter sequences were analyzed. As depicted in Fig 7A, the predicted RcsB binding element -KMRGAWTMWYCTGS- [58] could be identified within the regions of P_*hdeDB*_, P_*yfdX*_, P_*kvhAS*_, P_*hdeB1*_, P_*hdeA*_, and P_*hdeD1B2*_. The *rcsB* deletion effects on each promoter activity were then measured using LacZ as the reporter. As shown in Fig 7B, after the bacterial strain was grown to the exponential phase and then cultured at pH 4.4 for 1 h for acid adaptation, the promoter activity of *yfdX*, *hdeDB*, or *hdeB1* was reduced by the deletion of *rcsB*, whereas that of *kvhAS*, *hdeA*, or *hdeD1B2* exhibited no apparent changes (Fig 7B).

To determine whether RcsB directly regulates the expression of *yfdX* and *hdeDB*, electrophoretic mobility shift assay (EMSA) analysis was subsequently performed. As shown in Fig 7C, the recombinant RcsB could bind to the intergenic DNA between *yfdX* and *hdeDB* (P_*yfdX*_), or between *kvhAS* and *hdeB1* (P_*hdeB1*_), and the interaction could be inhibited by an excess amount of the nonlabeled probe P_*yfdX*_ or P_*hdeB1*_ demonstrating the binding specificity (Fig 7C). As depicted in the lower panel of Fig 7C, the interaction between His-RcsB and P_yfdX_* was also outcompeted by an excess amount of P_*hdeB1*_. Moreover, the binding between His-RcsB and P_yfdX_* or His-RcsB and P_hdeB1_* was interfered by the addition of acetyl phosphate further supporting that phosphorylation status of RcsB determines its regulatory role in influencing the expression of P_*yfdX*_, P_*hdeDB*_ and P_*hdeB1*_

### YfdX expression may be affected by the phosphorylated form of RcsB

**Fig 8:**
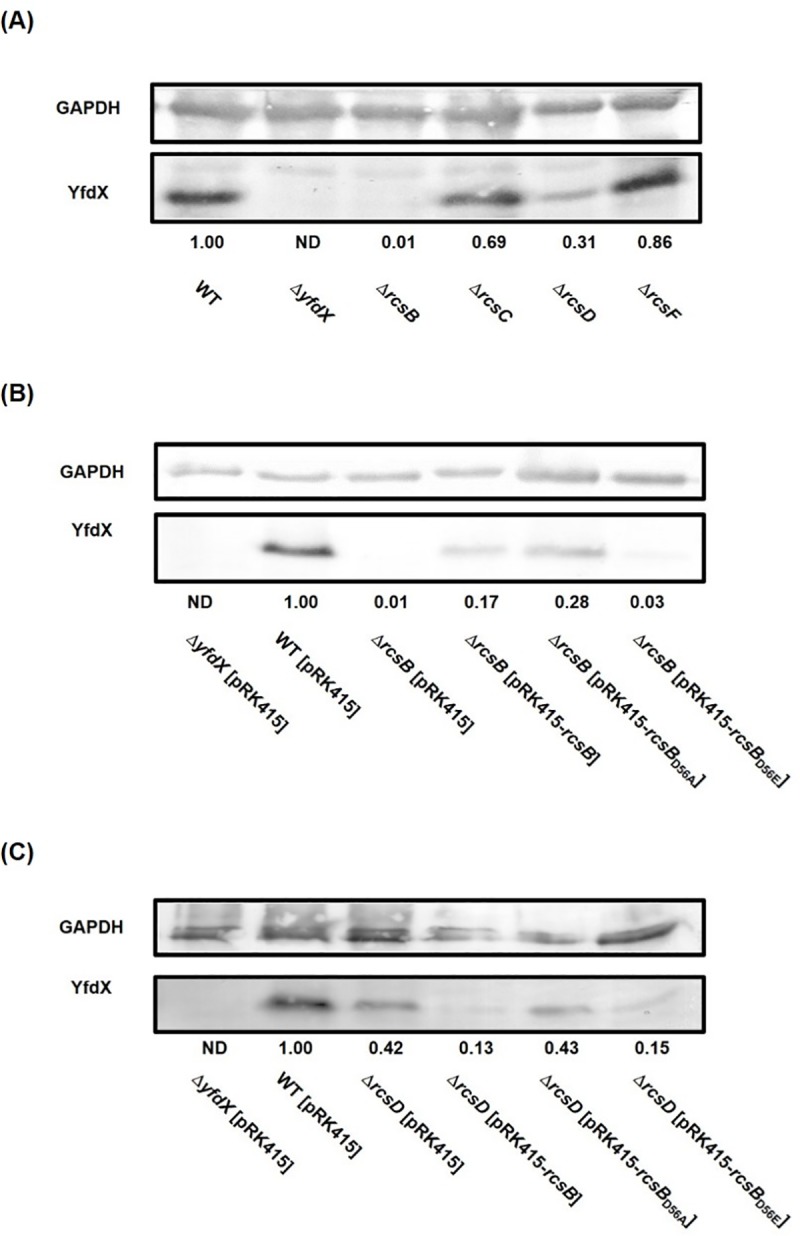
The phosphorylated form of RcsB influences the production of YfdX. (A) Western blot analysis for YfdX expression in Δ*rcsB*, Δ*rcsC*, Δ*rcsD*, and Δ*rcsF* strains. (B) Complementation analysis of the influences of RcsB phosphorylation status on YfdX production. (C) Analysis of the deleting effects of *rcsD* sensor kinase gene on the RcsB phosphorylation-dependent control. Bacteria was grown to the exponential phase and then cultured at pH 4.4 for 1 h for acid adaptation, and then total proteins were collected for western blot analysis of YfdX expression using anti-YfdX antiserum. The fold change of YfdX amount calculated using ImageJ software is shown. GAPDH was probed as protein loading control.

To investigate whether RcsB phosphorylation affects the acid response regulation as assessed by YfdX expression, Western blot analysis was conducted. Consistent with the promoter activity analysis, as shown in Fig 8A, the production of YfdX in the acidic LB broth (pH 4.4) was blocked by *rcsB* deletion but was unaffected by *rcsC* or *rcsF* deletion in both culture conditions. Notably, the YfdX production was reduced by the deletion of *rcsD*. And as shown in Fig 8B, the effect of *rcsB* deletion on YfdX production could be partly restored by complementation of RcsB and RcsB_D56A_, but not RcsB_D56E_. The phosphorylated form of RcsB (RcsB_D56E_) could not restore the production of YfdX.

To further confirm the involvement of RcsD in the acid response regulation, the *rcsD* deletion effect was also determined in the complementation analysis. As shown in Fig 8C, in the *rcsD* deletion mutant, overexpression of RcsB_D56A_ exhibited higher production of YfdX than that of RcsB or RcsB_D56E_. These findings suggest that RcsD may determine the nonphosphorylated form of RcsB, and RcsB in the nonphosphorylated form positively regulates the acid stress response.

## Discussion

A comparative analysis of the proteomes and the promoter activity analysis in *K*. *pneumoniae* CG43S3 demonstrated a positive control of YfdX expression by RcsB, which suggested that YfdX expression may be used as a reporter for the RcsB-mediated regulation of the acid response. The expression of *yfdX* is positively controlled by RcsB, and RcsD seems to be required to ensure that RcsB is in the nonphosphorylated form to activate the expression of YfdX. We have also demonstrated that YfdX as well as HdeA may function as periplasmic chaperones to protect *K*. *pneumoniae* CG43S3 from acid stress damage.

In *E*. *coli*, the nonphosphorylated form of RcsB positively affects the expression of AR2 [31, 32, 34]. Fig 2 illustrates that nonphosphorylated RcsB (RcsB_D56A_) but not phosphorylated RcsB (RcsB_D56E_) may complement the *rcsB* deletion effect. This suggests that nonphosphorylated RcsB (RcsB_D56A_) also plays a major role in the acid stress response of *K*. *pneumoniae* which lacks AR2. Proteome analysis revealed that several differential proteins regulated by RcsB were induced under acidic conditions. We isolated only spot 832 (YfdX) for further study because it exhibited significant changes. Nevertheless, we could not exclude the involvement or importance of these proteins that were not identified.

The *hdeA* containing gene cluster is only present in CG43 but not in the genome of NTUH-K2044 and MGH78578. We speculate that the gene cluster may have been horizontally acquired during evolution of CG43. As shown in Fig 4, *hdeA* deletion had no effect on acid stress survival. By contrast, *hdeA* overexpression enhanced the acid survival of both wild type (S5B Fig) and *yfdX* deletion mutant (Fig 5). This indicates that HdeA may exhibit a chaperone activity against acid stress in CG43S3. Nevertheless, we speculated that HdeA might not be expressed at exponential phase due to the repression of that in *E*. *coli* by H-NS [34, 52, 53], but the result revealed that HdeA was also unnecessary at stationary phase. when and how HdeA functions in the bacteria to respond to acid stress warrants further investigation. The results of acid survival analysis also indicated that HdeD and HdeB1 may play an important role in protecting CG43S3 against acid damage. As shown in S6 Fig, compared with Δ*hdeB*[pRK415-*hdeD*], Δ*hdeB*[pRK415-*hdeDB*] exhibited a higher level of acid survival. This implies a major role of HdeD while an assistant role of HdeB in AR. However, if HdeB assists HdeD in *K*. *pneumoniae* resistance to acid stress remains to be investigated.

The effect of *yfdX* deletion was cross-complemented by HdeA suggesting YfdX as well as HdeA functions as a chaperone (Fig 5). As shown in Fig 6, the result that the purified recombinant protein YfdX reduced the acid-induced protein aggregation further corroborated its chaperone activity. This is supported by a recent report indicating that STY3178 exhibits a periplasmic chaperone activity [56].

Acid survival analysis in Fig 2B suggested that RcsB in nonphosphorylated form played a positive role in the AR response. The possibility was further supported by the EMSA showing that adding acetyl phosphate interfered the binding efficiency of RcsB-P_yfdX_* or RcsB-P_hdeB1_*. As shown in Fig 8 and S7 Fig, the YfdX production in Δ*rcsB* strain could be induced by expression of RcsB or RcsB_D56A_, while that in Δ*rcsD* strain only be induced by RcsB_D56A_, suggesting that RcsD determines the nonphosphorylated form of RcsB. In *Salmonella*, a phosphoryl group could be transferred differentially from RcsC or RcsD to RcsB depending on specific stimuli [59]. Whether the sensor kinase RcsC is required for the phosphorelay in regulating the acid stress response remains unknown.

Deletion of *rcsB* or *rcsD* reduced the YfdX production at either pH 4.4 (Fig 8A) or pH 7 (S7A Fig). However, the deletion effect of *rcsB* or *rcsD* on YfdX production could be fully restored by expression of RcsB or RcsB_D56A_ in bacteria grown at pH 7 (S7B and S7C Fig) but could only be partly restored in bacteria grown in pH 4.4. Since the expression of YfdX, as assessed using promoter activity and protein production levels, was substantially higher in bacteria grown after pH 4.4 adaptation, we speculate that YfdX expression at pH 4.4 may be subjected to other regulatory system besides RcsBD.

In summary, this is the first report demonstrating that YfdX may be involved in the acid stress response as a periplasmic chaperone and the expression of *yfdX* is positively controlled by RcsB. Moreover, RcsD is required to ensure that RcsB is in the nonphosphorylated form to activate the expression of YfdX to enable acid stress response.

## Supporting information

S1 Fig AR comparison of *K*. *pneumoniae* CG43S3 and NTUH-K2044S3. Both bacteria were grown to the exponential phase (OD_600_ 0.6~0.7) or the stationary phase (OD_600_ 1.0~1.1) and treated with acid stress.
(TIF)

S2 Fig Acid survival analysis of deletion effect of *rcs genes* and complementation effect of nonphosphorylated or phosphorylated RcsB. Acid survivals of CG43S3, Δ*rcsB*, Δ*rcsC*, Δ*rcsD*, and Δ*rcsF* (A), and Δ*rcsB*[pRK415], Δ*rcsB*[pRK415-*rcsB*], Δ*rcsB*[pRK415-*rcsB*_D56A_], and Δ*rcsB*[pRK415-*rcsB*_D56E_] (B) are shown. The mutant and complement strains were grown to the exponential phase (OD_600_ 0.6~0.7). The samples were then diluted serially and dropped into LB agar plates, incubated at 37°C overnight.
(TIF)

S3 Fig Promoter activity analysis of *rcsDB* in different pH conditions. The promoter activity was assessed by monitoring the expression of β-galactosidase on the plasmid pLacZ15 cloned with the promoter regions of *rcsDB* on Δ*lacZ* strains. Bacteria grown to the exponential phase were resuspended in the LB broth (pH 7.0, pH 5.5, and pH 4.4) for 1 h and then measured the promoter activity. Error bars indicate standard deviations of three independent experiments done in triplicate.
(TIF)

S4 Fig Acid survival analysis of the deletion effect of *yfdX* and *hde* genes. The *hde* genes include *hdeB*, *hdeD*, *hdeDB*, *hdeB1*, *hdeA*, *hdeB2*, and *hdeD1B2*. The mutant strains were grown to the exponential phase (OD_600_ 0.6~0.7) (A) or stationary phase (OD_600_ 1.0~1.1) (B) and treated with acid stress. The samples were then diluted serially and dropped into LB agar plates, incubated at 37°C overnight.
(TIF)

S5 Fig Acid survival analysis of the complementation effect of *yfdX* and *hde* genes. (A) Complementation effects of YfdX, HdeB, HdeD, HdeDB, HdeA and HdeA_F44A_ on Δ*yfdX* strain. (B) overexpression effect of HdeA on wild type strain. The strains were grown to the exponential phase and treated with acid stress. The samples were then diluted serially and dropped into LB agar plates, incubated at 37°C overnight.
(TIF)

S6 Fig Complementation analysis of the *hdeB* deletion effect. Plasmid pRK415 carrying gene coding for HdeB, HdeD, or HdeDB were individually transformed into Δ*hdeB* strain, and the resulting complement strains were grown to the exponential phase (OD600 0.6~0.7) and treated with acid stress and acid survival analysis was performed.
(TIF)

S7 Fig Western blot analysis of the *rcs* genes deletion and complementation effect on YfdX production under normal condition. (A) Western blot analysis for YfdX expression in Δ*rcsB*, Δ*rcsC*, Δ*rcsD*, and Δ*rcsF* strains. (B) Complementation analysis of the influences of RcsB phosphorylation status on YfdX production. (C) Analysis of the deleting effects of *rcsD* sensor kinase gene on the RcsB phosphorylation-dependent control. Bacteria was cultured in LB broth (pH 7) at 37°C for 20h, and then total proteins were collected for western blot analysis of YfdX expression using anti-YfdX antiserum. The fold change of YfdX amount calculated using ImageJ software is shown. GAPDH was probed as protein loading control.
(TIF)

S1 Table Analysis of the spots which exhibited differences between the proteomes of CG43S3 and CG43S3Δ*rcsB*. (DOCX)
