# Supplementary figures and images for: RcsB regulation of the YfdX-mediated acid stress response in Klebsiella pneumoniae CG43S3
Source: PLoS One. 2019 Feb 28;14(2):e0212909. doi: 10.1371/journal.pone.0212909 (PMC6394985; doi:10.1371/journal.pone.0212909)

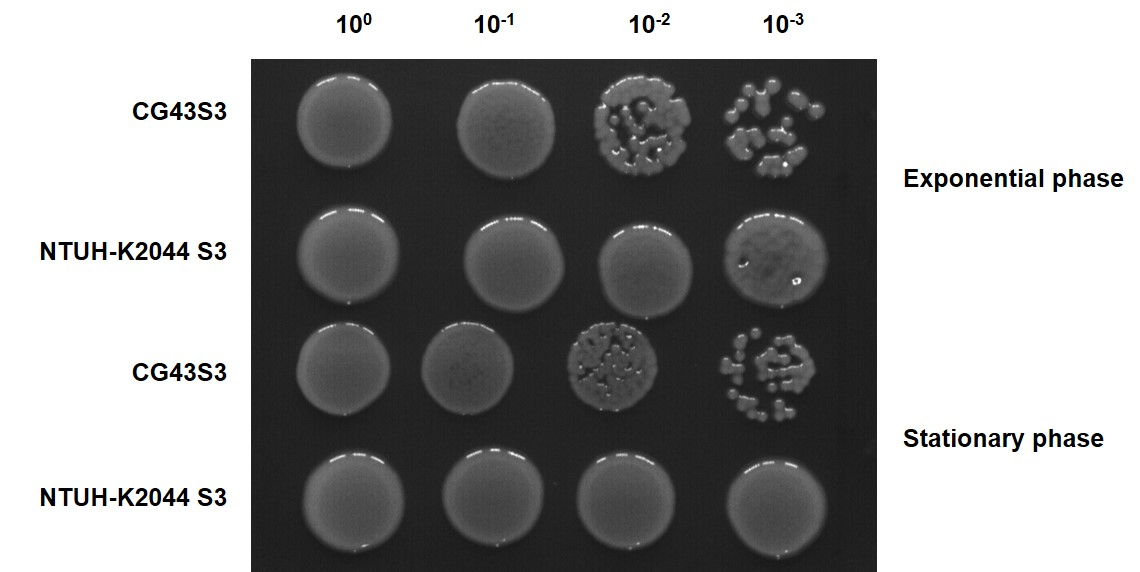

Supplement: S1 Fig — Both bacteria were grown to the exponential phase (OD600 0.6~0.7) or the stationary phase (OD600 1.0~1.1) and treated with acid stress. (TIF) [file pone.0212909.s001.tif]

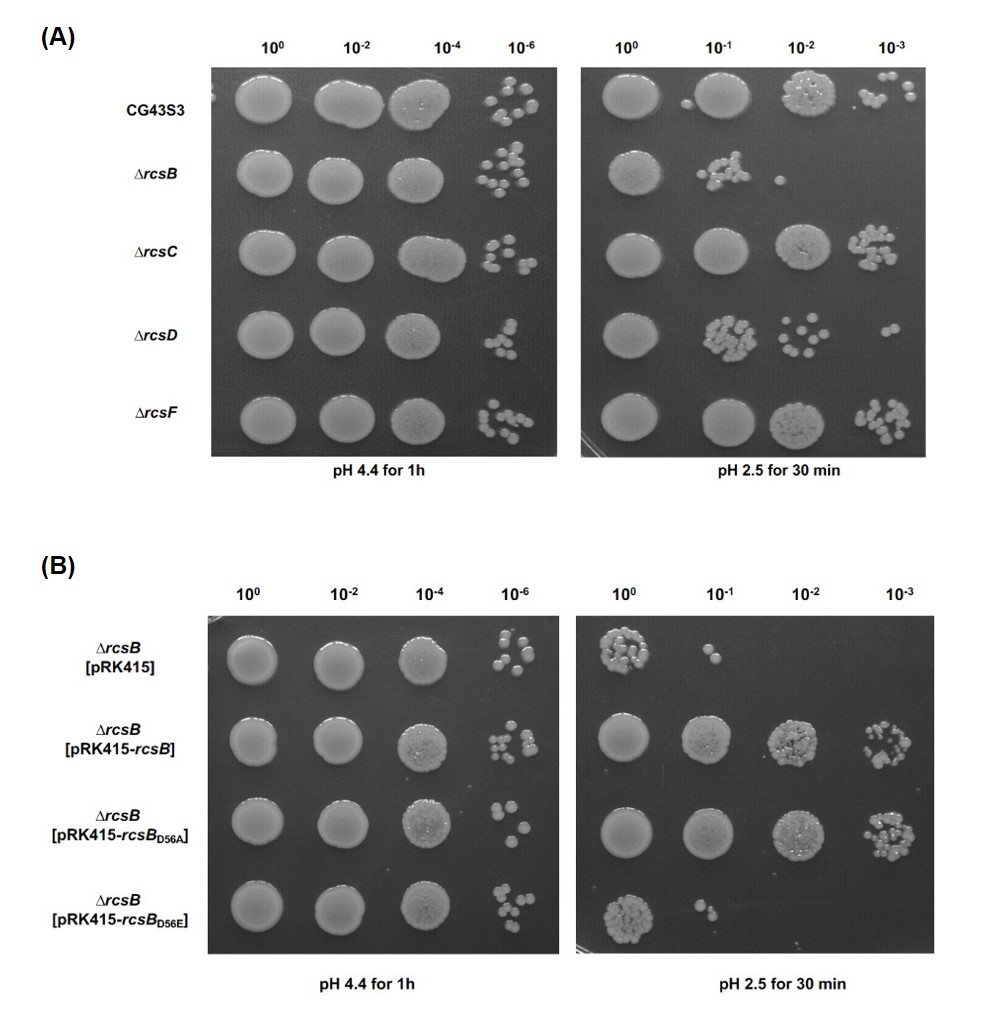

Supplement: S2 Fig — Acid survivals of CG43S3, ΔrcsB, ΔrcsC, ΔrcsD, and ΔrcsF (A), and ΔrcsB[pRK415], ΔrcsB[pRK415-rcsB], ΔrcsB[pRK415-rcsBD56A], and ΔrcsB[pRK415-rcsBD56E] (B) are shown. The mutant and complement strains were grown to the exponential phase (OD600 0.6~0.7). The samples were then diluted serially and dropped into LB agar plates, incubated at 37°C overnight. (TIF) [file pone.0212909.s002.tif]

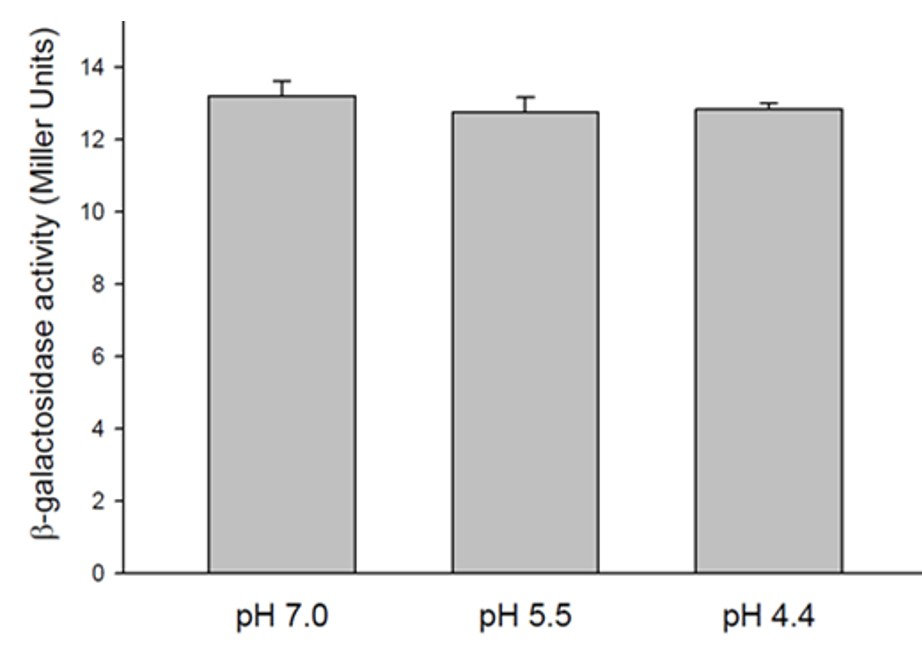

Supplement: S3 Fig — The promoter activity was assessed by monitoring the expression of β-galactosidase on the plasmid pLacZ15 cloned with the promoter regions of rcsDB on ΔlacZ strains. Bacteria grown to the exponential phase were resuspended in the LB broth (pH 7.0, pH 5.5, and pH 4.4) for 1 h and then measured the promoter activity. Error bars indicate standard deviations of three independent experiments done in triplicate. (TIF) [file pone.0212909.s003.tif]

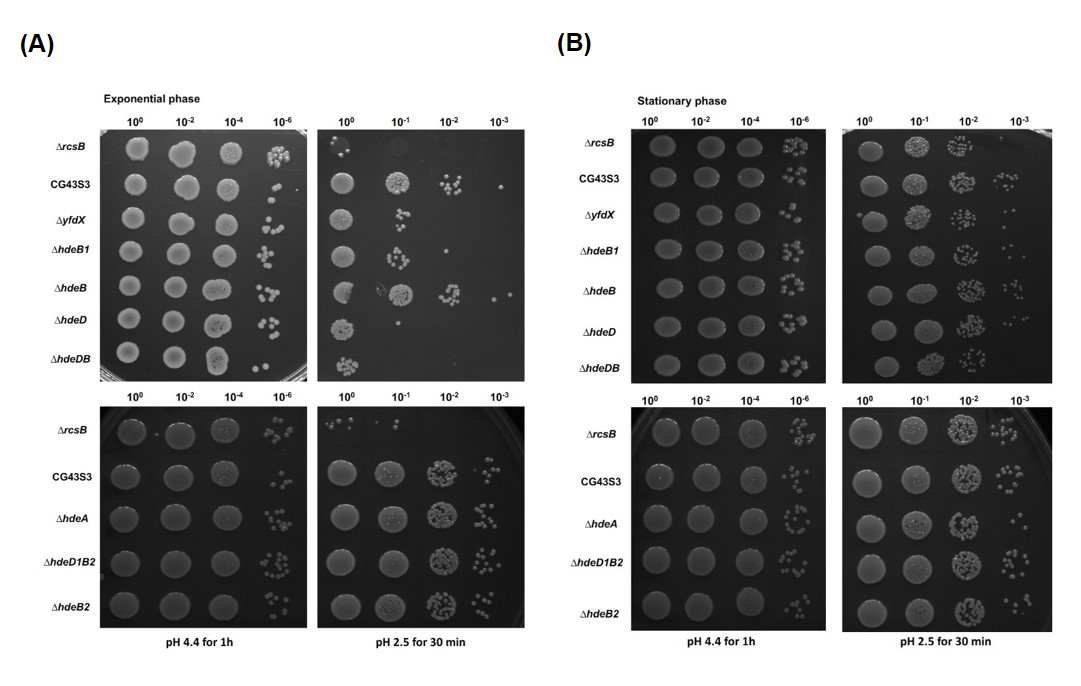

Supplement: S4 Fig — The hde genes include hdeB, hdeD, hdeDB, hdeB1, hdeA, hdeB2, and hdeD1B2. The mutant strains were grown to the exponential phase (OD600 0.6~0.7) (A) or stationary phase (OD600 1.0~1.1) (B) and treated with acid stress. The samples were then diluted serially and dropped into LB agar plates, incubated at 37°C overnight. (TIF) [file pone.0212909.s004.tif]

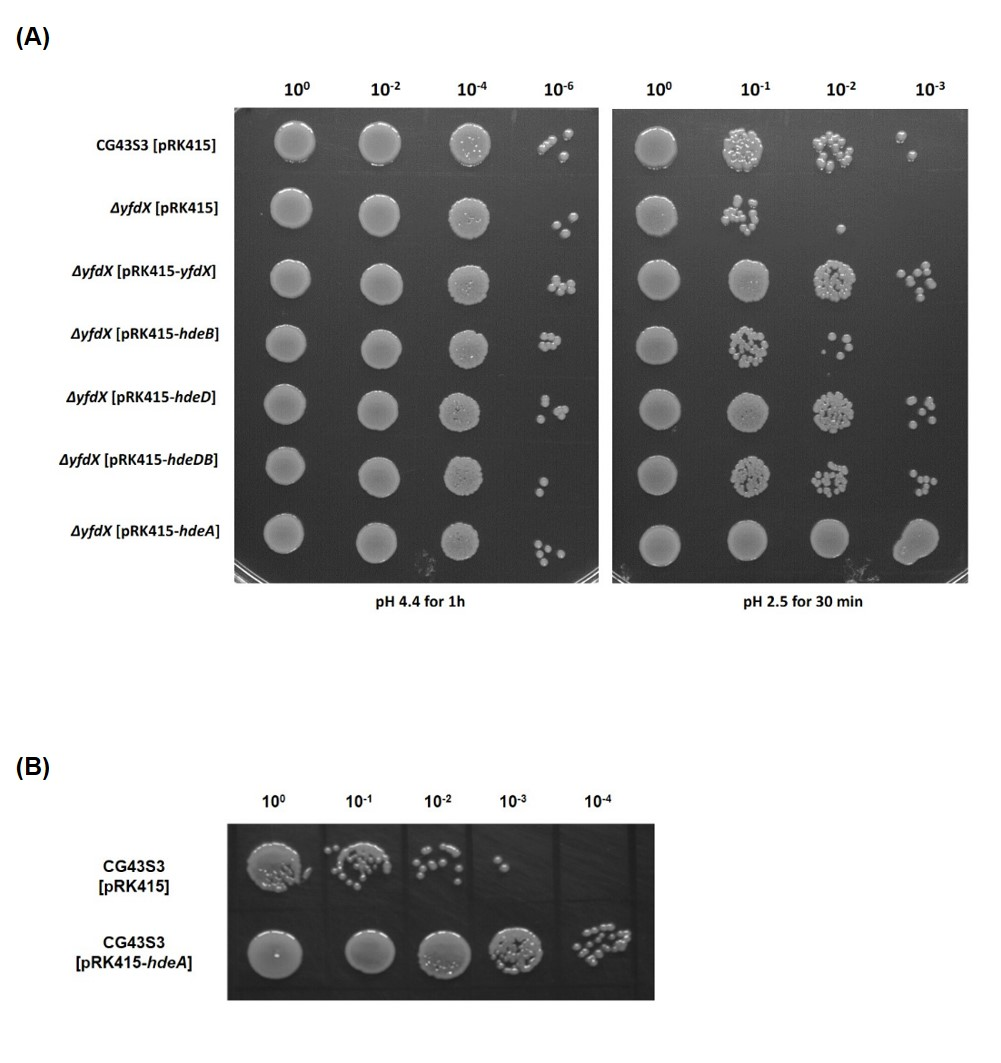

Supplement: S5 Fig — (A) Complementation effects of YfdX, HdeB, HdeD, HdeDB, HdeA and HdeAF44A on ΔyfdX strain. (B) overexpression effect of HdeA on wild type strain. The strains were grown to the exponential phase and treated with acid stress. The samples were then diluted serially and dropped into LB agar plates, incubated at 37°C overnight. (TIF) [file pone.0212909.s005.tif]

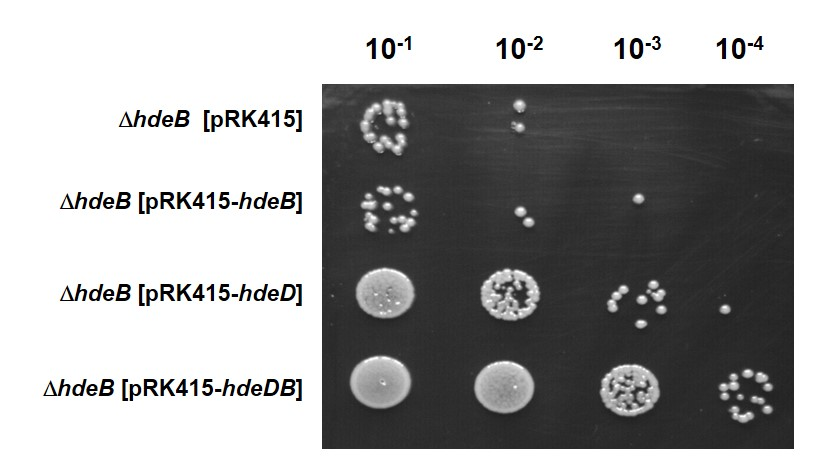

Supplement: S6 Fig — Plasmid pRK415 carrying gene coding for HdeB, HdeD, or HdeDB were individually transformed into ΔhdeB strain, and the resulting complement strains were grown to the exponential phase (OD600 0.6~0.7) and treated with acid stress and acid survival analysis was performed. (TIF) [file pone.0212909.s006.tif]

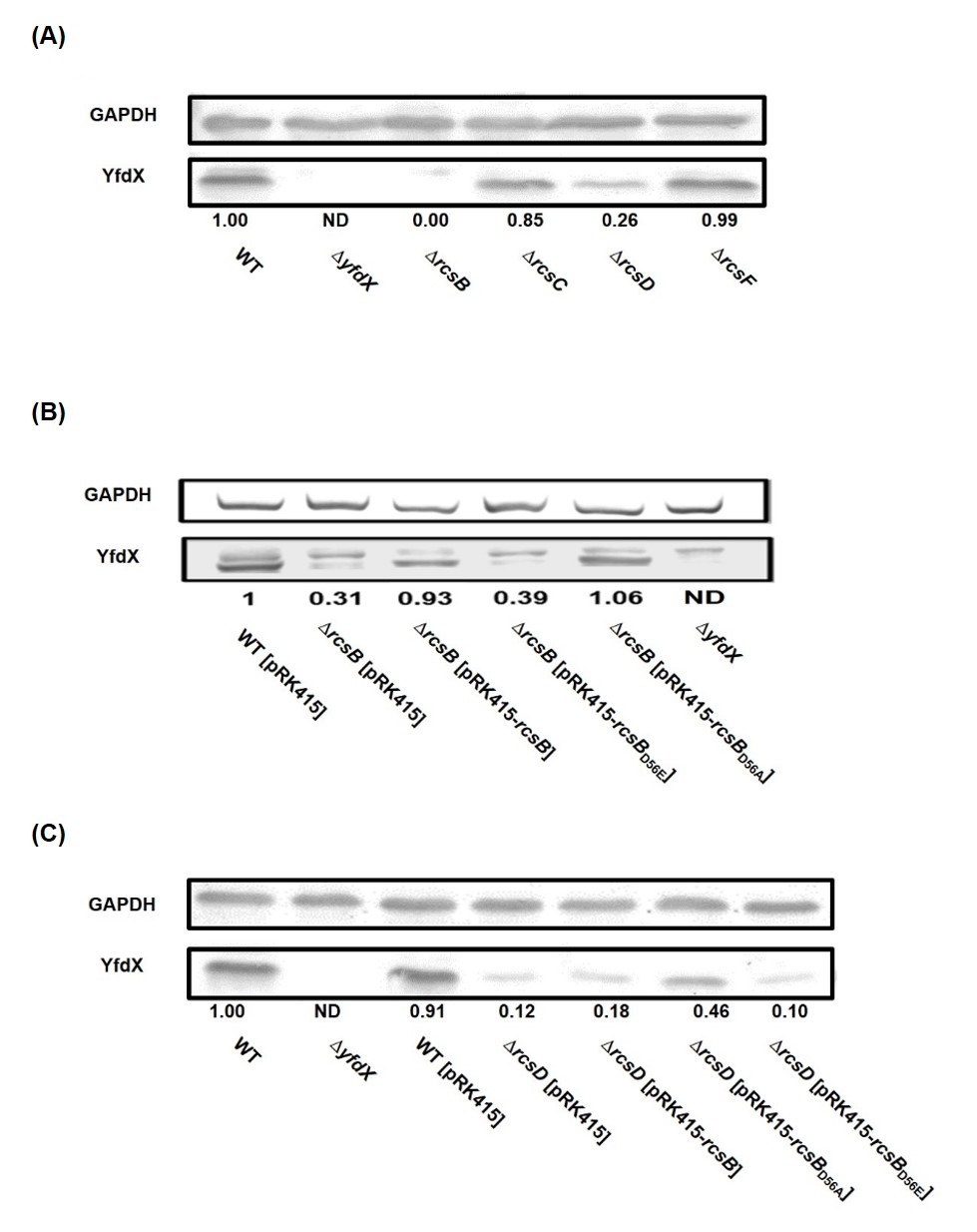

Supplement: S7 Fig — (A) Western blot analysis for YfdX expression in ΔrcsB, ΔrcsC, ΔrcsD, and ΔrcsF strains. (B) Complementation analysis of the influences of RcsB phosphorylation status on YfdX production. (C) Analysis of the deleting effects of rcsD sensor kinase gene on the RcsB phosphorylation-dependent control. Bacteria was cultured in LB broth (pH 7) at 37°C for 20h, and then total proteins were collected for western blot analysis of YfdX expression using anti-YfdX antiserum. The fold change of YfdX amount calculated using ImageJ software is shown. GAPDH was probed as protein loading control. (TIF) [file pone.0212909.s007.tif]
