# Supplementary material for: RcsB regulation of the YfdX-mediated acid stress response in Klebsiella pneumoniae CG43S3
Source: PLoS One. 2019 Feb 28;14(2):e0212909. doi: 10.1371/journal.pone.0212909 (PMC6394985; doi:10.1371/journal.pone.0212909)
Supplement: S1 Table — (DOCX) [file pone.0212909.s008.docx]

**S1 Table. Analysis of the spots which exhibited differences between the proteomes of CG43S3 and CG43S3Δ*rcsB*.**

| **Match ID** | **pI** | **mw (kDa)** | **Expressional fold change** |
| --- | --- | --- | --- |
| **772** | **5.98** | **25** | **↓2.18** |
| **817** | **5.23** | **21** | **─_a_** |
| **832** | **5.05** | **19** | **─_a_** |
| **879** | **5.92** | **14** | **↓1.13** |
| **946** | **5.98** | **10** | **↓1.22** |
| **972** | **4.70** | **9** | **↓1.90** |
| **973** | **4.59** | **9** | **↓1.52** |
| **1064** | **5.08** | **18** | **↑1.14** |
| **a：protein which had no detection in Δ*rcsB* strain** | | | |
